# Trilliumosides K and L, two novel steroidal saponins from rhizomes of *Trillium govanianum*, as potent anti-cancer agents targeting apoptosis in the A-549 cancer cell line

**DOI:** 10.3389/fchem.2023.1306271

**Published:** 2023-12-22

**Authors:** Bashir Ahmad Lone, Misbah Tabassum, Anil Bhushan, Dixhya Rani, Urvashi Dhiman, Ajaz Ahmad, Hilal Ahmad Mir, Prem N. Gupta, D. M. Mondhe, Sumeet Gairola, Prasoon Gupta

**Affiliations:** ^1^ CSIR-Human Resource Development Centre, Academy of Scientific and Innovative Research, Ghaziabad, India; ^2^ Natural Products and Medicinal Chemistry Division, CSIR-Indian Institute of Integrative Medicine, Jammu, India; ^3^ Pharmacology Division, CSIR-Indian Institute of Integrative Medicine, Jammu, India; ^4^ Department of Clinical Pharmacy, College of Pharmacy, King Saud University, Riyadh, Saudi Arabia; ^5^ Department of Ophthalmology, Pathology, and Cell Biology, Columbia University, New York, NY, United States; ^6^ Plant Science and Agrotechnology Division, CSIR-Indian Institute of Integrative Medicine, Jammu, India

**Keywords:** *Trillium govanianum*, saponins, steroidal glycosides, A-549, BAX, BCL-2, cytotoxicity

## Abstract

Two novel steroidal saponins, trilliumosides K (**1**) and L (**2**), were isolated from the rhizomes of *Trillium govanianum* led by bioactivity-guided phytochemical investigation along with seven known compounds: govanoside D (**3**), protodioscin (**4**), borassoside E (**5**), 20-hydroxyecdysone (**6**), 5,20-hydroxyecdysone (**7**), govanic acid (**8**), and diosgenin (**9**). The structure of novel compounds 1-2 was established using analysis of spectroscopic data including 1D and 2D nuclear magnetic resonance (NMR) and high resolution mass spectrometry (HR-ESI-MS) data. All isolated compounds were evaluated for *in vitro* cytotoxic activity against a panel of human cancer cell lines. Compound **1** showed significant cytotoxic activity against the A-549 (Lung) and SW-620 (Colon) cancer cell lines with IC50 values of 1.83 and 1.85 µM, respectively whereas the IC50 value of Compound **2** against the A-549 cell line was found to be 1.79 µM. Among the previously known compounds **3**, **5**, and **9**, the cytotoxic IC50 values were found to be in the range of 5–10 µM. Comprehensive anti-cancer investigation revealed that Compound **2** inhibited *in vitro* migration and colony-forming capability in the A-549 cell line. Additionally, the mechanistic analysis of Compound **2** on the A-549 cell line indicated distinctive alterations in nuclear morphology, increased reactive oxygen species (ROS) production, and decreased levels of mitochondrial membrane potential (MMP). By upregulating the pro-apoptotic protein BAX and downregulating the anti-apoptotic protein BCL-2, the aforementioned actions eventually cause apoptosis, a crucial hallmark in cancer research, which activates Caspase-3. To the best of our knowledge, this study reports the first mechanistic anti-cancer evaluation of the compounds isolated from the rhizomes of *T. govanianum* with remarkable cytotoxic activity in the desired micromolar range.

## Graphical Abstract

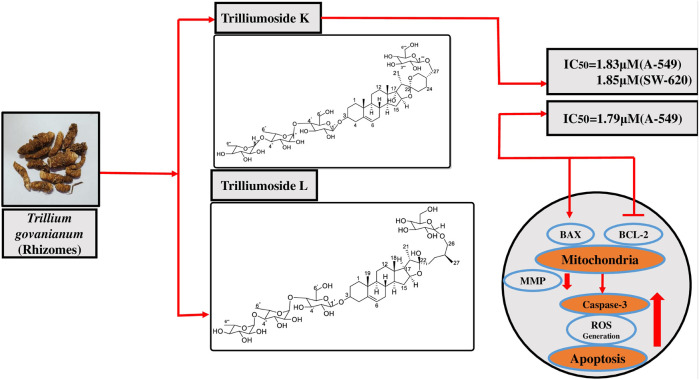

## Highlights

1) Extraction, isolation and characterization of two new steroidal saponins using detailed NMR studies along with seven known compounds from bioactive fraction of *Trillium govanianum.*
2) Evaluate *in vitro* cytotoxic potential of *T. govanianum* extracts, fractions and purified compounds against various human cancer cell lines.
3) Compound **1** showed significant cytotoxic activity against A-549 (Lung) and SW-620 (Colon) cell lines with IC_50_ values of 1.83 and 1.85 µM, whereas compound (2) IC_50_ value against A-549 cell line was found to be 1.79 µM.
4) Compound 2 demonstrated significant cytotoxic effect by inhibiting cell proliferation, promoting apoptosis in lung (A-549) carcinoma.
5) Our study reports the first mechanistic anticancer evaluation of the compounds isolated from the rhizomes of *T. govanianum* with remarkable activity in the desired micro molar range.

## 1 Introduction

*Trillium govanianum* (TG) is an indigenous, perennial medicinal herb of the North-Western Himalayan region belonging to the family Melanthiaceae. Genus *Trillium* consists of 42 species found in different areas across the globe. According to the Global Biodiversity Information Facility (GBIF), most of the species are located in North America and Europe, and only nine are found in the Asian continent (Ohara and Kawano, 2005; Sofi et al., 2022). In India, TG is commonly known by vernacular names such as Nag Chhatri and is distributed in an altitudinal range of 2,500–3,800 m in the Himalayan region (Chauhan et al., 2019; Sharma and Samant, 2104). In the traditional medicine system, the rhizomes of TG are used to treat wound healing, skin diseases, dysentery, and menstrual and sexual disorders (Pant and Samant, 2010; Rani et al., 2013). Increased market demand for TG at the international level is due to an essential phytosteriod sapogenin, i.e., Diosgenin (2.5%), which is an important component of commercial steroids and sex hormones (Sharma et al., 2018). Previous phytochemical investigations of the genus *Trillium* have reported the identification of fatty acid esters, saponins, phenolics, terpenoids, flavonoids, and steroids. Among all the isolates, steroidal saponins were found to be major bioactive compounds (Ono et al., 1986; Mimaki and Watanabe, 2008; Huang and Zou, 2011; Ur Rahman et al., 2017; Yan et al., 2021). To date, more than 30 steroidal saponins have been discovered from *Trillium* species and they were found to exhibit anti-oxidant, anti-fungal, anti-inflammatory, and anti-cancer properties (Yan et al., 2009)**.**

**FIGURE 1:**
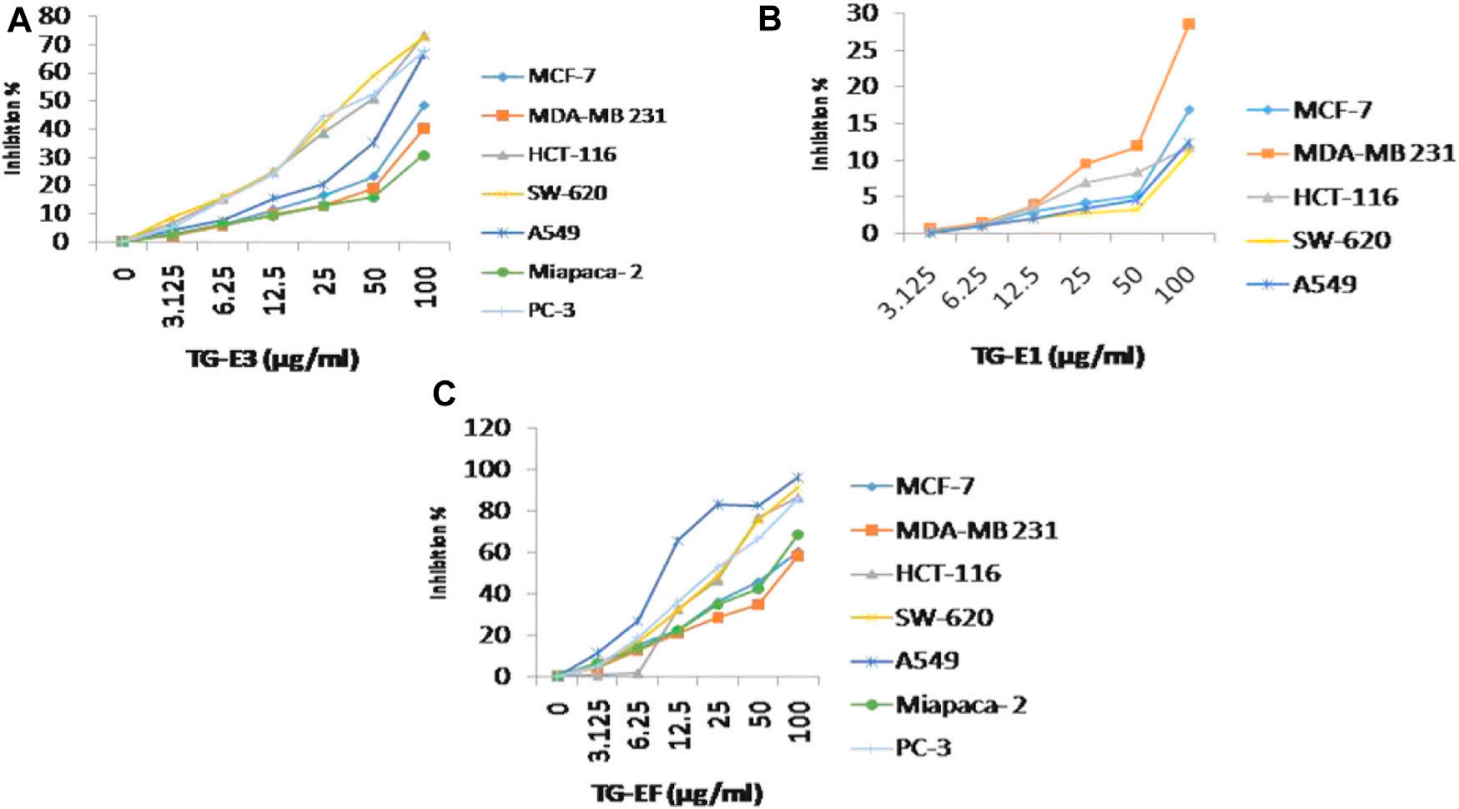
Cytotoxicity activity (% growth inhibition) for **(A)** TG-E1, **(B)** TG-E3, and **(C)** TG-EF at different concentrations and µg/mL.

**FIGURE 2:**
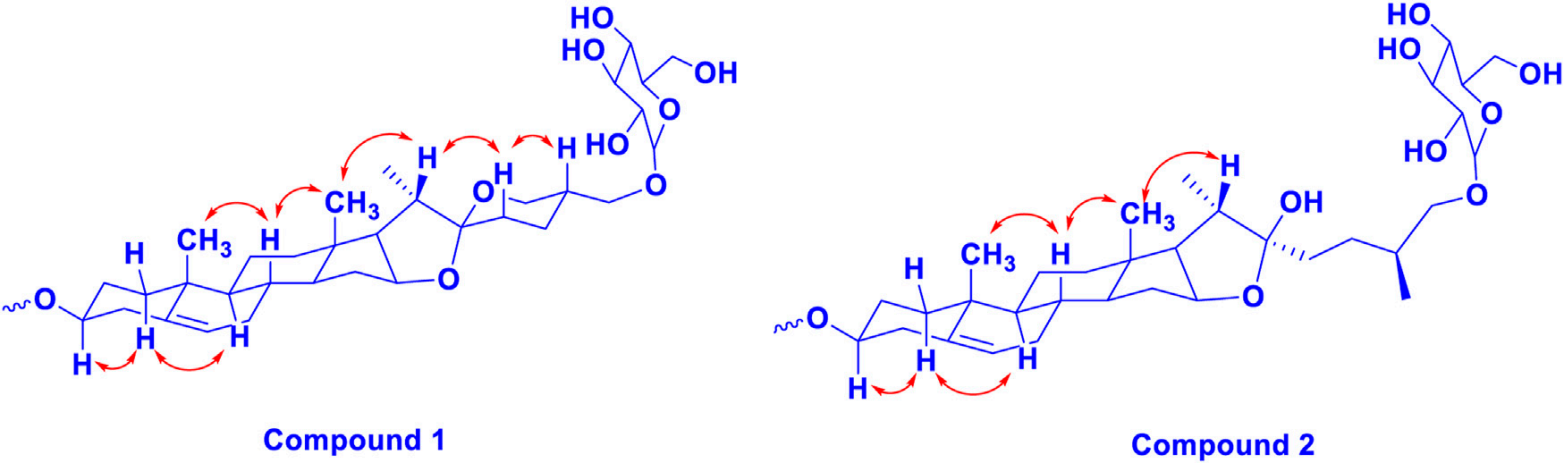
The key ^1^H-^1^H NOESY (arrow) correlations of **1** and **2**.

Cancer is one of the most difficult public health issues that humanity has ever faced. Cancer develops because of aberrant cells proliferating uncontrollably, which has the potential to invade and damage healthy bodily tissues. The disease is characterized by high rates of morbidity and mortality; in many countries, it ranks second in terms of cause of death, after cardiovascular disease. Chemotherapy, either administered alone or in conjunction with radiotherapy and surgery, is currently the primary cancer treatment modality used to lower the death rate from the disease globally (Kumar et al., 2023). Although a number of therapeutic agents have been effectively tested and employed to eradicate various forms of cancer, adverse effects have consistently been demonstrated to be significant issues (Beniwal et al., 2022). Certainly, there is an immense requirement to create novel, potent, and efficient new medicinal entities that have improved effectiveness and fewer negative consequences. Throughout history, natural products have been the most important source of drug molecules. Approximately 74% of anti-cancer drugs are natural products or derived from natural products, highlighting their major role in cancer chemotherapy (Tan et al., 2006). Steroidal saponins are important natural glycosidic compounds that possess an amphiphilic character and show remarkable cytotoxic activity (Ya-Zheng et al., 2018). Recent studies have shown that saponins can inhibit the growth of numerous cancer cells, via their ability to decrease tumor growth, induce apoptosis, promote autophagy, and control the tumor microenvironment via several signaling pathways (Wang et al., 2018; Zhang et al., 2019). Plant also exhibits antibacterial, anti fungal, anti informatory and anti oxidant properties (Ismail et al., 2015). In our continuing efforts to discover cytotoxic compounds in plants, the crude extracts, fractions. The present study aimed to isolate and characterize two new steroidal saponins (**1–2**) and seven known compounds (**3–9**) (Figures 1, 2). Structural determination of these compounds was based on modern spectroscopic analysis, including high resolution mass spectrometry (HRMS), one dimensional (1D), two dimensional (2D) nuclear magnetic resonance (NMR), and acid hydrolysis.

## 2 Material and methods

### 2.1 General experimental procedures and chemicals used

Column chromatography was done by using silica gel (60–120 and 100–200 mesh size), HP-20, HP-20SS, Sephedex, and silver nitrate-incorporated silica gel. Merck Kieselgel (Aufoilen) 60 F254 plates were used for thin-layer chromatography (TLC). High resolution mass spectra were obtained on Agilent 6540 (Q-TOF) mass spectrometer, in the electrospray (ESMS) mode. All solvents used for high performance liquid chromatography (HPLC) analysis were obtained from Merck Chemicals (Mumbai India). Water used for extraction and isolation purposes was obtained at Central Drug House (P) Ltd., Delhi. Spectroscopic data (NMR) of isolated compounds was gathered on a ^1^H NMR at 400 and 500 MHz and on a^13^C NMR at 100 and 125 MHz (Bruker Advance spectrometer). The reference point was TMS (*δ*_H_ and *δ*_C_: 0.00 ppm). Chemical shifts (*δ*) were referenced internally to the residual solvent peak (CD_3_OD: ^1^H- 3.30, ^13^C- 49.0 ppm). In 2D NMR, all heteronuclear ^1^H and ^13^C correlations were established based on gradient-enhanced inverse-detected Heteronuclear Multiple Bond Correlation (HMBC) and Heteronuclear single quantum coherence (HSQC) experiments. HP-20, HP-20SS, Sephedex, silver nitrate, dimethylsulphoxide (DMSO), and all other chemicals were purchased from Sigma–Aldrich (St. Louis, MO, United States).

### 2.2 Plant material

The rhizomes of *T. govanianum* were collected from (the Shroth Dhar, Paddar Kisthwar) area of Distt. Doda (J&K, India) at the altitude of 3,000–3,074 masl. Authentication of the crude herb was done by Dr. Sumeet Gairola (Plant Science and Agrotechnology Division IIIM), and the voucher specimen (No. RRLH-23418) was submitted to the Crude Drug Repository of CSIR-IIIM, Jammu.

### 2.3 Extraction and isolation

**FIGURE 3:**
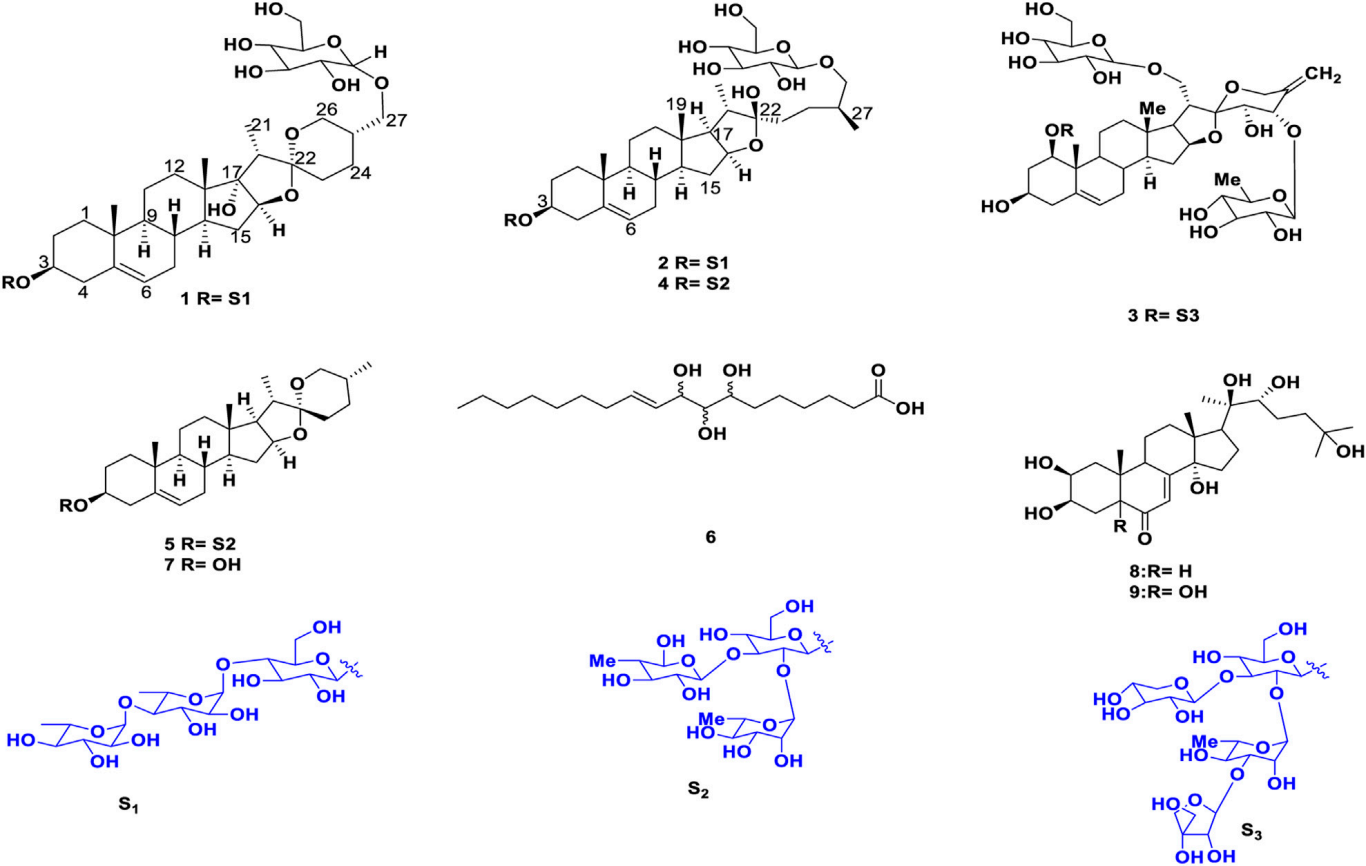
Structures of the identified compounds (1–9).

Shade-dried rhizomes of *T. govanianum* (1.9 kg) were ground to powder and sequentially extracted twice (drenched for 12 h each) with 3.5 L of chloroform followed by 3 L H2O: MeOH (20:80) thrice. The extracts were filtered separately and concentrated using rota vapor at 45°C at reduced pressure to obtain crude chloroform and hydroalcoholic extract (187.78 g and 229.44g), respectively. Further (20% aq. MeOH) extract (229.44 g) was directly subjected to reverse phase column chromatography for purification using HP-20 dianion exchange resin eluted with a gradient of water: methanol solvent system (100:00.0–10:90.0 one lit er collected measurements of all fractions) and on concentration, giving 20 collective subfractions (Fr.HA.01-Fr.HA.20). Further, white precipitate was formed in (Fr.HA.12-Fr.HA.16), which on washing with HPLC grade water and separated using Whatman filter paper grade-1 yielded white amorphous powder **compound 5** (8.67 g). The isolate was detected on TLC [CHCl_3_: MeOH: H2O (6.5:3:0.5)] as a single dark green spot upon heating the dried TLC in the anisaldehyde sulphuric acid reagent. Polar subfractions (Fr.HA.01-Fr.HA.07) (26.45 g) with the same chemical profile were pooled and processed for further purification on HP-20SS resin, using a gradient of MeOH: H2O (95%–55%), which afforded 35 small fractions (100 mL each), and these fractions were divided into six subfractions on the bases of their chemical profiling, namely, Fr.1A-Fr.1F. Fraction Fr.1C (2.24 g) upon purification using reverse phase dianion (HP-20SS) eluted with a gradient of water: methanol solvent system of 0.5:9.5–8.5:2.5 25 ml each collected in 50 mL test tubes, based on the TLC profile fractions 6.5:3.5 and 8.5:2.5, afforded **compound 1 and 2** (27.11, and 17.75 mg) as a white amorphous solid and dark brown crystalline solid, respectively. Furthermore, purification of fraction F-1A-1B (1.95 g) on HP-20SS, using an H2O: MeOH solvent system (70:30), afforded **compound 3** (40 mg) as a white solid powder. Remixing (95:0.5–75:2.5) fractions (0.87 g) and again purifying using dianion (HP-20SS) resin, eluted with an isocratic solvent system of MeOH: H2O (75:25), yielded **compound 4** (61 mg) as a yellowish powder. Chloroform extract (187.78 g) was processed for fractionation to separate the oily non-polar part using solvent–solvent fractionation. Extract (187.78 gm) was dissolved in 1 L of CHCl_3,_ 800 mL of water was added to solubilize the extract, and an equal amount of cyclohexane was used. After partition, the hexane layer was separated and concentrated using rotavapor (36.11 gm) with an oily mass left behind. The hexane fraction/extract was processed for purification by normal phase column chromatography (CC) (silver nitrate incorporated silica gel, 100–200 mesh) to isolate lipid compounds using a distilled hexane:ethyl acetate solvent system for elution (100:0 to 70:30, 100 mL fractions were collected). These were concentrated using rotavapor, giving a total of 11 pooled fractions (Fr.H.1–Fr.H.11) based on their TLC outline. The fraction (Fr.H.6-Fr.H.9) gave a single major spot after charring and drying in the anisaldehyde reagent and was washed with chilled cyclopentane 100 mL to obtain **compound 6** (White powder 2.66 gm). The remaining chloroform residual extract (131.66 gm) was processed for the isolation of individual compounds by CC (silica gel, 100–200 mesh), and eluted with hexane:ethyl acetate (500 mL collected volumes) with increasing polarity. The eluted fractions were concentrated on rotavapor, giving six new fractions (Fr.CR-1– Fr.CR-6) based on the TLC analysis. Fraction CR-03 (17.78 g) was again processed for purification by CC using silica gel 100–200 mesh with n-hexane-ethyl acetate (1:0 to 8:2, 100 mL volumes were collected) to obtain nine daughter fractions (Fr.3A-Fr.3I). Upon placing these fractions overnight on working table, colorless needles were formed in Fr.3D-Fr.3G. This yielded **compound 7** (1.11g), which was visualized using a developing solvent system of Hex-EtOAc (80:20); a bright green spot appeared after spraying anisaldehyde reagent and heating the TLC plate. Moreover, Fr.CR.4-Fr.CR.6 (28.45 g) fractions were again purified by CC on silica gel (60–120 mesh, 200 g), using a gradient of CHCl_3_–MeOH (100%–80%), and a total of 22 fractions were obtained (100 mL each). These fractions were separated into six subfractions (Fr.A1-Fr.A6) based on similarities in their TLC profiles. The several times repeated CC of F.B2 fraction (7.27 g) on silica (230–400 mesh, 100 g), using the increasing polarity of CHCl_3_–MeOH (85:15), afforded **compounds 8** and **9** (0.9 gm) and (42 mg), respectively. The chemical structures of all the isolated and characterized compounds are presented in Figure 3.

**Trilliumoside K (1):** Brownish solid (27.11 mg). Its molecular formula was determined to be C_51_H_85_O_23_, by ESI-MS data at m/z 1064.5568 [M + H]^+^ (calcd. for C_51_H_85_O_23_^+^,1064.53), together with its NMR data (Figure 3) (Tables 1, 2).

**TABLE 1:** ^1^H and ^13^C NMR spectroscopic data for aglycone of trilliumosides K and L (**1** and **2)** ^a^.

Position	1		2	
	*δ*_H_ (Int, mult, *J* in Hz)	*δ*_C_	*δ*_H_ (Int, mult, *J* in Hz)	*δ*_C_
1a b	1.09 (1H, *m*)	38.67	1.06 (1H, *m*)	38.68
	1.82 (1H, *m*)		1.87 (1H, *m*)	
2a b	1.91 (1H, *m*)	30.55	1.90 (1H, *m*)	31.48
	1.62 (1H, *m*)		1.65 (1H, *m*)	
3	3.53 (1H, *m*)	79.45	3.39 (1H, *m*)	79.93
4a b	2.29 (1H, *brd*, 12.2 Hz)	39.58	2.43 (1H, *m*)	39.60
	2.44 (1H, 12.2, 2.4 Hz)		2.27 (1H, *m*)	
5	—	141.95	—	142.00
6	5.38 (1H, *brd*)	122.75	5.38 (1H, *brd*)	122.75
7a b	1.98 (1H, *m*)	32.33	2.00 (1H, *m*)	33.29
	1.35 (1H, *m*)		1.53 (1H, *m*)	
8	1.54 (1H, *m*)	33.18	1.63 (1H, *m*)	32.88
9	0.94 (1H, *m*)	51.52	0.91 (1H, *m*)	51.87
10	—	38.01	—	38.15
11a b	1.55 (1H, *m*)	21.18	1.55 (1H, *m*)	22.07
	1.51 (1H, *m*)		1.54 (1H, *m*)	
12a b	1.91 (1H, *m*)	30.55	1.57 (1H, *m*)	30.86
	1.23 (1H, *m*)		1.91 (1H, *m*)	
13	—	46.12	—	46.61
14	1.68 (1H, *m*)	53.92	1.12 (1H, *m*)	57.87
15a b	1.55 (1H, *m*)	33.01	1.57 (1H, *m*)	29.07
	1.98 (1H, *m*)		1.98 (1H, *m*)	
16	4.11 (1H, *dd*, 6.1, 3.2)	90.89	4.36 (1H, *dd*, 6.1, 3.2)	82.53
17	—	91.62	1.72 (1H, *m*)	65.14
18	0.86 (3H, *s*)	17.50	0.82 (3H, *s*)	20.01
19	1.36 (3H, *s*)	20.10	1.04 (3H, *s*)	17.44
20	2.38 (1H, *m*)	50.00	2.15 (1H, *m*)	41.93
21	0.92 (3H, *s*)	9.87	0.83 (3H, *s*)	16.97
22	—	112.46	—	114.10
23a b	1.69 (1H *m*)	36.88	1.73 (1H, *m*)	40.94
	1.67 (1H *m*)		1.83 (1H, *m*)	
24a b	1.18 (1H, *m*)	28.55	1.14 (1H, *m*)	28.75
	1.68 (1H, *m*)		1.60 (1H, *m*)	
25	1.73(1H, *m*)	35.07	1.73 (1H, *m*)	35.08
26a b	3.41 (1H, *m*)	58.43	3.38 (1H, *m*)	76.12
	3.72 (1H, *m*)		3.70 (1H, *m*)	
27a b	3.37 (1H, *m*)	76.11	1.73 (3H, *m*)	26.11
	3.70 (1H, *m*)			

^a^ Recorded in CD_3_OD, at 500 MHz (^1^H) and 100 MHz (^13^C) with TMS, as internal standard, chemical shifts, multiplicity and coupling constants (*J*, Hz) were assigned by means of ^1^H and ^13^C NMR, spectral data.

**TABLE 2:** ^1^H and ^13^C NMR spectroscopic data for glycone of trilliumosides K and L (**1** and **2)** ^a^.

Position	1		2	
	*δ*_H_ (Int, mult, *J* in Hz)	*δ*_C_	*δ*_H_ (Int, mult, *J* in Hz)	*δ*_C_
3-O-Glc-1*′*	4.49 (1H, *d*, 7.8)	*100.50*	4.49 (1H, *d*, 7.8)	100.54
2*′*	3.24 (1H, *m*)	77.95	3.25 (1H, *m*)	77.95
3*′*	3.38 (1H, *m*)	78.16	3.59 (1H, *m*)	78.16
4*′*	3.55 (1H, *m*)	79.92	3.53 (1H, *m*)	79.92
5*′*	3.73 (1H, *m*)	76.69	3.66 (1H, *m*)	76.69
6*′*	4,15 (1H, *dd*, 12.8, 6.7) 3.79^b^	62.00	3.66 (1H, *dd*, 12.4, 6.3) 3.79^b^	62.04
2-O-Rha-1*″*	4.85 (1H, *br.s*)	103.4	4.83 (1H, *br.s*)	103.07
2*″*	3.27 (1H, *m*)	71.75	3.26 (1H, *m*)	71.75
3*″*	3.40 (1H, *m*)	73.98	3.39 (1H, *m*)	73.98
4*″*	3.39 (1H, *m*)	79.32	3.55 (1H, *m*)	79.32
5*″*	4.12 (1H, *m*)	69.86	4.12 (1H, *m*)	69.86
6*″*	1.24 (3H, *d*, 6.4)	18.14	1.24 (1H, *m*)	18.14
4-O-Rha-1*‴*	5.19 (1H, *br.s*)	102.2	5.19 (1H, *br.s*)	102.42
2*‴*	3.77 (1H, *m*)	72.50	3.64 (1H, *m*)	72.50
3*‴*	3.65 (1H, *m*)	72.25	3.83 (1H, *m*)	72.25
4*‴*	3.41 (1H, *m*)	73.81	3.40 (1H, *m*)	73.81
5*‴*	3.93 (1H, *m*)	70.72	3.92 (1H, *m*)	70.72
6*‴*	1.27 (3H, *d*, 6.4)	18.02	1.25 (1H, *m*)	18.01
26-OGlc-1*′′′′*	4.23 (1H, *d*, 7.8)	104.66	4.23 (1H, *d*, 7.8)	104.67
*2′′′′*	3.12 (1H, *m*)	75.24	3.12 (1H, *m*)	75.24
3*′′′′*	3.38 (1H, *m*)	78.19	3.34 (1H, *m*)	78.19
4*′′′′*	3.84 (1H, *m*)	72.40	3.82 (1H, *m*)	72.40
5*′′′′*	3.59 (1H, *m*)	78.04	3.51 (1H, *m*)	78.04
6*′′′′*	(a) 3.64 (1H, *m*)^b^	62.83	(a) 3.91, (1H, *m*)^b^	62.91
	(b) 3.68 (1H, *m*)^b^		(b) 3.82 (1H, *m*)^b^	

^a^ Recorded in CD_3_OD, at 500 MHz (^1^H) and 100 MHz (^13^C) with TMS, as internal standard, chemical shifts, multiplicity, and coupling constants (*J*, Hz) were assigned using ^1^H and ^13^C NMR, spectral data.

^b^ Overlapped with other signals can be interchanged.

Mutliplicity of NMR signals is written in Italics.

**Trilliumoside L (2):** This was isolated as a brownish amorphous solid (17.75 mg). Its molecular formula was determined to be C_51_H_85_O_22_, by HR-ESI-MS data at m/z 1049.5413 [M + H]^+^ (calcd. for C_51_H_85_O_22_^+^, 1049.55), together with its NMR data (Tables 1, 2).

**Govanoside D (3):** Yellow amorphous powder, (37 mg); ESI-MS (Negative): 1356.60 (M-H)^-^ (Calcd. C_61_H_95_O_33_^−^, 1356.60); the ^1^H NMR (400 MHz, CD_3_OD) and ^13^C NMR (100 MHz, CD_3_OD) data agreed with literature data of Govanoside D (Singh et al., 2021).

**Protodioscin (4):** White colored amorphous solid, (40 mg); ESI-MS: (Positive) *m/z* 1071.43 [M + Na]^+^ (Calcd. for C_51_H_84_O_22_Na^+^, 1071.43); the ^1^H-NMR (CD_3_OD, 400 MHz) and ^13^C-NMR (CD_3_OD, 100 MHz) experimental data agreed with literature data of Protodioscin (Abdel-Sattar et al., 2008).

**Borassoside E (5):** White colored amorphous solid, (8.67 gm); ESI-MS (Negative): 867.46 (M-H)^-^ (Calcd. 867.48, C_45_H_71_O_16_^−^); the ^1^H NMR (400 MHz, CD_3_OD) and ^13^C NMR (100 MHz, CD_3_OD) experimental data agreed with that of literature data of Borassoside E (Yokosuka and Mimaki, 2008a; Ismail et al., 2015).

**Govanic acid (6):** White solid, (1.11 gm) ESI-MS (Positive); 331.2436 (M + H)^+^ (calcd., C_18_H_35_O_5_^+^, 331.2436**)**; the ^1^H NMR (400 MHz, CDCl3) and ^13^C NMR (100 MHz, CDCl3) data was found to be identical to literature data of Govanic acid (Ur Rahman et al., 2017).

**Diosgenin (7):** Colorless crystalline, solid (678 mg); ESI-MS (Positive): 415 (M + H)^+^ (Calcd. 415.31, C_27_H_43_O_3_^+^); the ^1^H NMR (400 MHz, CDCl_3_) and ^13^C NMR (100 MHz, CDCl3) data resembled that of literature data of Diosgenin (Ismail et al., 2015).

**20-Hydroxy ecdysone (8):** White colored solid**,** (0.9g) HR-MS *m/z* 481.3161 [M + H] ^+^(calcd for C_27_H_45_O_7_^+^, 481.3161). the ^1^H NMR (400 MHz, CD_3_OD) and ^13^C NMR (100 MHz, CD_3_OD) observed data agreed with previously reported literature data of 20-Hydroxy ecdysone (Ur Rahman et al., 2017).

**5,20-Hydroxy ecdysone (9):** White colored solid**,** (40 mg) ESI-MS (Positive); 497.30 (M + H)^+^ (calcd. C_18_H_45_O_8_^+^, 497.31); the ^1^H NMR (400 MHz, CDCl_3_), and ^13^C NMR (100 MHz, CDCl_3_) data was found to be similar to previously reported data of 5,20-Hydroxy ecdysone (Ur Rahman et al., 2017).

### 2.4 Sugar analysis of compounds (1–2)

#### 2.4.1 Acid hydrolysis and GC/MS analysis

Both new compounds, **1** and **2** (10 mg), were each dissolved in 5 mL MeOH and then 5 mL 5% HCl (V/V) was added to the solution. The reaction mixture was refluxed in an oil bath at 100°C for 3 h after the completion of the reaction. To separate the aglycone component, methanol was distilled, and the reaction mixture was agitated with CHCl_3_. Using rotavapor, the CHCl_3_ extract was dried over anhydrous sodium sulfate, and the solvent was then distilled out. The residue was dissolved in HPLC-grade methanol before undergoing aglycone moiety analysis. The silver oxide was used to neutralize the acidic aqueous mother liquor, and the precipitate that resulted was filtered out and washed three times with distilled water. The combined filtrate and washings were concentrated using a rotary evaporator under reduced pressure at 50°C. Pyridine and acetic anhydride were used to further acetylate the residue, and G.C. grade EtOAC was used to prepare the acetylated sample for GC/MS analysis. By comparing the sugar units' retention times to those of the reference sugar, the sugar units from the G.C. analysis were verified (acylated). The presence of 32.705 (*β*-D-glucose) and 26.202 (*α*-L-rhamnose) was confirmed by GC/MS analysis, given in S.I. (Supplementary Figures S9, S18), and additional sugar units were discovered based on the NMR data (Love and Simons, 2020).

### 2.5 Biology

#### 2.5.1 Cell culture and growth conditions

Different human cancer cell lines, such as lung (A-549; HOP-62), breast (MCF-7; MDA-MB 231), pancreatic (Mia PaCa-2), colon (SW-620; HCT-116), prostate (PC-3), and neuroblastoma (SH-SY5Y) were grown in growth medium (RPMI-1640 and DMEM) boosted with 10% fetal bovine serum (FBS Qualified: Standard origin Brazil 10270106), streptomycin (100 units/mL) and penicillin (100 units/mL) in tissue culture flasks. fR2 is a normal cell line and was grown in RPMI-1640 media supplemented with 10% FBS and maintained in a CO_2_ incubator (Thermocon Electron Corporation, United States) at 37°C with permissible atmospheric conditions of (95%) air and (5%) CO_2_ with (98%) humidity. These cell lines were procured from the National Cancer Institute (NCI), United States.

#### 2.5.2 Sulphorhodoamine B (SRB) assay

The SRB assay is a colorimetric assay performed to evaluate the cytotoxic potential of active inhibitors (Desai et al., 2008). Ninety six well flat transparent plates were seeded with the optimal cell density per well (Flat bottom). 100 µL/well of cell suspension of each cell line was plated with their specific cell number, such as PC-3 (7000), MCF-7 (8000), MDA-MB 231 (7500), A-549 (7500), Mia PaCa-2 (6500), SW-620 (7500) HCT-116 (7000), HOP-62 (7500) and allowed to grow overnight at 37^°^C with 5% Co_2_ in a cell-culture incubator. The cells were treated with various concentrations of test compounds (1, 5, 10, 30, and 50 µM) after 24 h of incubation, along with camptothecin as a positive control. The cells were incubated again at similar culture conditions for 48 h, and ice-cold TCA fixed the cells for 1 h at 4^°^C. Plates were washed thrice with water and allowed to dry. Further, at room temperature, 0.4% SRB dye was added (100 µL in each well) for half an hour. The plate was then washed thrice with water and once with 1% v/v acetic acid to remove the unbound SRB and allowed to dry for some time at room temperature. To solubilize the bound dye, 100 µL of 10 mM Tris buffer (pH–10.4) was added to each well. The plates were then kept in the shaker for 5 min to dissolve the protein-bound dye properly. Finally, the Optical Density (OD) was noted at 540 nm in a microplate reader, and then IC_50_ was calculated using GraphPad Prism Software.

$$\text{The }\%\text{ of cell viability}=\frac{\text{Absorbance}\;\text{of}\;\text{treated}\;\text{cells}\;-\;\text{Absorbance}\;\text{of}\;\text{Blank}}{\text{Absorbance}\;\text{of}\;\text{control}\;\text{cells}-\text{Absorbance}\;\text{of}\;\text{Blank}}\times 100$$

% Growth inhibition=100 − % of cell viability

#### 2.5.3 DAPI staining

A-549 cells were seeded in six-well plates (1 × 10^5^) for 24 h and treated with various Compound 2 concentrations for 48 h. After 48 h, PBS wash was given, cell fixation was done using cold methanol, and plates were kept at 4^°^C for 20 min. Next, the cells were stained with DAPI at 1 μg/mL concentration in PBS. The morphological alterations in the cell nuclei were observed using a fluorescence microscope, specifically the Olympus IX53. This microscopy allowed for the visualization and assessment of changes in the structure of the cell nuclei induced by the treatment.

#### 2.5.4 Reactive oxygen species (ROS) assay

A-549 cells cell was seeded at the density (2 × 10^5^) for 24 h in a 6-well plate. After 24 h of incubation, different concentrations of Compound **2** for 48 h were used and 0.05% H_2_O_2_ was used as a positive control. After the PBS wash, cell staining was done using DCFH-DA (Dichlorodihydro-fluorescein diacetate) dye. The reactive oxygen species (ROS) levels were subsequently analyzed using a fluorescence microscope (Olympus IX53). This process allowed for the observation and quantification of the levels of ROS within the cells, providing insights into the oxidative stress induced by the treatment with Compound **2** and the positive control H_2_O_2_.

#### 2.5.5 Colony formation assay

A-549 cells were seeded at the density of 2 × 10^5^ for 24 h in 6-well plates. After 24 h of incubation, the cells were treated with Compound **2** at concentrations of 1, 2, 4, and 6 µM. Trypsinization of cells was done after 48 h, and cells were re-seeded as 1000 cells/well. Cells were allowed to regrow to form colonies to test the clonogenic potential. Fixation of cells was done with 4% formaldehyde (1 mL/well) followed by PBS wash. Staining was done with 0.5% crystal violet dye, and the colonies were counted (Vichai and Kirtikara, 2006).

#### 2.5.6 *In vitro* cell migration assay

A-549 cells were seeded in six well-flat transparent plates and allowed to confluent up to 70%–80% for 24 h in a starving condition (serum starved), and a straight horizontal line with a sterile 200 µL tip was created by scraping the monolayer of A-549 cells. Finally, the cells were treated with Compound **2** at concentrations of 1, 2, 4, and 6 µM for 48 h. Images of the wounded area were taken at 0 and 48 h, and the following equation demonstrated the percentage of wound closure:

$$\%\text{ Wound closure}=\frac{\left[1\;–(\;\text{wound}\;\text{area}\;\text{at}\;0\;h\right]}{\left[\;\text{wound}\;\text{area}\;\text{at}\;48h\;\right]}\times 100\;\%$$

#### 2.5.7 Mitochondrial membrane potential

Six-well plates were used for the culture of A-549 cells (1.5 × 10^5^) for 24 h and were treated with Compound 2 at concentrations of 1, 2, 4, and 6 µM and with 0.25 µM of camptothecin for 48 h. One hour before terminating the experiment, 10 nM Rhodamine −123 dye was added, and a PBS wash was given. Cells were analyzed under a fluorescence microscope (Olympus IX53) (Gupta et al., 2017a).

#### 2.5.8 Western blot analysis

For Western blotting, A-549 cells were seeded at a density of 3 × 10^3^ cells/well in six-well plates. After 24 h, the A-549 cells were treated with different concentrations of Compound **2**. The concentrations were 1, 2, 4, and 6 µM for 48 h. After 48 h, cells were washed with cold PBS. Cell lysates were prepared in 1X RIPA buffer (Sigma) with added sodium orthovanadate (100 mM), protease cocktail (Roche), NaF (100 mM), PMSF (100 mM), and EDTA (100 mM). Protein estimation was done using Bradford reagent (Bio-Rad). The samples were boiled with sample buffer containing 1% *β*-mercaptoethanol, 6% glycerol, 2% SDS, 22 mM Tris-HCl pH-6.8, and bromophenol blue. Whole-cell lysates corresponding to 70–100 ug of protein were loaded. SDS-PAGE analyzed the samples with a 10% separating gel (Gupta et al., 2017b). After running the gel, the proteins were transferred to the PVDF membrane and then blocked for 1 h in a solution of 5% BSA, 0.1% Tween 20, 150 mM NaCl, and 20 mM Tris-HCl pH-7.4. After blocking, the membrane was probed with primary BAX, BCL-2, and cleaved caspase-3, which was further incubated with the corresponding (CST) HRP-conjugated secondary antibodies. *β*-Actin was used as a gel loading control (Majeed et al., 2014).

#### 2.5.9 Statistical analysis

Data analysis was done using Microsoft Excel and GraphPad Prism 5 software. All data analysis was done using GraphPad Prism-5 software and ImageJ. The statistical significance of data was determined by one-way analysis of variance (ANOVA) and was accepted at *p* < 0.05.

## 3 Results and discussion

### 3.1 Chemistry

Crude chloroform and 20% aq. MeOH extract of the rhizomes of *T. govanianum* underwent a sequence of column chromatographic purification steps using silica gel, HP-20, HP-20SS resins, and Sephadex respectively, to obtain two new steroidal saponins, named trilliumoside K (**1**) and L (**2**) along with seven known previously reported compounds. Comparison of their NMR and MS data with the reported literature confirmed the structures of seven known compounds as govanoside D (Singh et al., 2021), protodioscin (Abdel-Sattar et al., 2008; Gupta et al., 2017b), borassoside E (Yokosuka and Mimaki, 2008a; Ismail et al., 2015), diosgenin (Ismail et al., 2015), govanic acid (Ur Rahman et al., 2017), 20-hydroxyecdysone (Ur Rahman et al., 2017), and 5,20-hydroxyecdysone (Ur Rahman et al., 2017). Moreover, the 1D and 2D NMR characterization data of both the new compounds are described.

**FIGURE 4:**
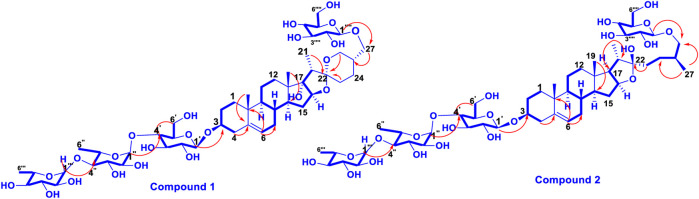
The important HMBC (→) and ^1^H-^1^H COSY (─) correlations (1 and 2).

**Trilliumoside K (1)** was isolated as a brownish solid. Its molecular formula, C_51_H_85_O_23_, was deduced by ESI-MS data at *m/z* 1064.5568 [M + H]^+^ (calcd for C_51_H_84_O_23_, 1063.52), along with its ^1^H and ^13^C NMR data (Tables 1, 2). The NMR spectra of compound **1** (TG-07B3) exhibited two typical angular signals for two quaternary methyls, resonating at *δ*_H_ 0.86 and 1.03 assignable to the CH_3_-18 (*s*) and CH_3_-19 (*s*) methyl groups; one methyl doublet for secondary methyl group at *δ*_H_ 0.92 (*d, J*, 6.3 Hz, 21-CH_3_); four oxymethylene protons at *δ*_H_ 3.41 (*m*, H-26a), *δ*_H_ 3.72 (*m*, H-26b), *δ*_H_ 3.37 (*m*, H-27a), and *δ*_H_ 3.70 (*m*, H-27b), (Supplementary Figure S1); and their respective ^13^C signals were found resonating at *δ*_C_ 58.43 and 76.12, with one olefinic proton resonating at *δ*_H_ 5.38 (*br., s*, H-6). In ^13^C there were two olefinic signals observed at *δ*_C_ 141.95 (C-5) and 122.75 (C-6) (Supplementary Figure S2) and one quaternary carbon characteristic of hemiketal functionality was found resonating at *δ*_C_ 112.46 (C-22). The downfield shift of *δ*_H_ at 3.53 and *δ*_C_ at 79.45 indicated the presence of hydroxyl or glycosidic substitutions at this position. The positions of protons were confirmed by using 2D HSQC and ^1^H-^1^H COSY spectra. Further, the ^13^C spectrum confirms the methine carbon at *δ*c 90.89 assigned to C-16 and 91.62 for quaternary carbon assigned to C-17 (Supplementary Figure S3) which indicated the presence of hydroxyl group at C-17 and the basic skeleton of aglycone was characteristic of pennogenin moiety when compared with literature data (Ju and Jia, 1992). The final stereochemistry of the steroidal skeleton was deduced based on nuclear Overhauser effect spectroscopy (NOESY) correlations. The cross peaks of H-1a on the NOESY spectrum with respect to H-3 were examined to determine the relative configuration of aglycone moiety. The NOESY (Supplementary Figure S7) correlations between H-8 and methyl groups at H-18 and H-19 confirmed the methyl groups to be β -oriented. Furthermore, the NOESY correlations between H-9/H-14, and H-14/H-16 (Figure 2) confirmed the ring fusion of the B\C and C/D rings was identified as trans and the D/E ring was found to be cis (Mimaki et al., 2008). The ^1^H NMR spectrum of compound **1** also revealed the presence of four characteristic anomeric proton signals found resonating at *δ*_H_ 4.23 (*d, J* = 7.8 *Hz*, *O*-Glc C-26), 4.49 (*d, J =* 7.8 Hz, *O*-Glc1-H-1), 4.85 (*br s*, Rha-1), and 5.18 (*br s*, Rha-2) (Supplementary Figure S1) while the corresponding anomeric carbon signals were assigned as *δ*_C_ 104.66, 100.5, 103.04, and 102.5 in ^13^C NMR spectra which was confirmed by HSQC spectra (Supplementary Figure S4). The ^13^C NMR spectrum exhibited a total of 51 carbon resonances where 27 signals corresponded to the aglycone part and the remaining 24 carbon resonances were assigned to the sugar units with four six-membered monosaccharide units. Moreover, the comparison of the NMR of compound **1** with that of trikamsteroids showed that they share the identical spirostanol skeleton of (*25R*)-5en-spirost-3 β **,**17 α ,27-triol, which was further confirmed by closer examination of 2D NMR data consisting of HSQC, HMBC, COSY and NOESY spectra of compound 1 (Yokosuka and Mimaki, 2008a)**.** The site of attachment of different sugar units was determined by their key HMBC correlations (Figure 4). The key HMBC correlation of Glc-H-1 (*δ*_H_ 4.49) with C3 (*δ*_C_ 79.45) of aglycone confirmed Glc-1*′* to be attached at C-3 of the aglycone moiety. There was another HMBC correlation observed between C-4*′* of Glc1*′* (*δ*_H_ 4.49) and Rha-H-1*′′* (*δ*_H_ 4.85) established Rha-1*″* unit to be attached at C-4 of Glc1*′*. Further, the close examination of the HMBC spectrum showed the correlation between C-3 of Rha-H-1*′′* (*δ*_C_ 79.32) with H-1*‴* of Rha-2 (*δ*_H_ 5.18), confirming that the site of attachment of Rha-2 to be at C-3 of Rha1. There was another correlation between O-Glc-H*‴* (*δ*_H_ 4.23) and C-26 (*δ*_C_ 76.11). The *β*-orientation of both D-glucopyranosyls was confirmed by their relatively large *J*-values of anomeric protons (7.8 *Hz*, *O*-Glc C-26 and 7.8 *Hz*, *O*-Glc1-H-1, respectively). In addition to the NMR data, the presence of sugar units and their orientation was further confirmed by acid hydrolysis of **compound 1** which resulted in the liberation of two units each of *D*-glucose and *L*-rhamnose. It was done by relating their ^1^H and ^13^C NMR data and coupling constants with the literature reports. Based on above data, the structure of **compound 1** was assigned as 27-*O-β-D*-glucopyranosyl-(25R)-5-en-spirost-3*β*,17*α*,27-triol-3-*O-α-*L-rhammopyranosyl-(1–4)-[*α-L*-rhamnopyranosyl-(1–4)] *-β-D-*glucopyranoside (Trilliumoside K).

**Trilliumoside L (2)** was obtained as a yellow-brownish solid. Its molecular formula was determined to be C_51_H_85_O_22_, by HR-ESI-MS data at *m/z* 1049.5413 [M + H]^+^ (calcd. for C_51_H_84_O_22_, 1048.55), in combination with ^1^H and ^13^C NMR data (Tables 1, 2). The analysis of the ^1^H and ^13^C NMR spectral data of compound **2** revealed the presence of two typical angular signals for two quaternary methyls resonating at *δ*_H_ 0.82 and 1.04, assignable to the CH_3_-18 (s) and CH_3_-19 (s) methyl groups, and two methyl doublets for two secondary methyl groups at *δ*_H_ 0.96 (*d*, *J*, 6.2 *Hz*, CH_3_-27) and 1.20 (*d, J*, 6.3 *Hz*, 21-CH_3_) (Supplementary Figure S10). There was also a characteristic signal for two oxymethylene protons observed at *δ*_H_ 3.32 (m, H-26a) and *δ*_H_ 3.73 (m, H-26 b), and an olefinic proton resonating at *δ*_H_ 5.38 (*br*, *s*, H-6). In ^13^C NMR data, two olefinic signals were found resonating at *δ*_C_ 141.95 (C-5) and 122.75 (C-6) while there was one characteristic quaternary hemiketalic carbon at *δ*_C_ 114.46 (C-22). Furthermore, it was confirmed that the configuration of a hydroxyl group at C-22 was identified as *β* indicated by its resonance at *δ*_C_ 114.46, instead of an *α* configuration for which *δ*_C_ 112 is the characteristic resonance (Yokosuka and Mimaki, 2008a). The configuration 25 *R* was confirmed by the chemical shift difference between the geminal protons H-26a (*δ* 3.38, m) and H-26b (*δ* 3.70, m) (Δ *δ*ab = 0.32 ppm): Δ *δ*ab < 0.48 ppm for 25*R* and Δ δab >0.57 ppm corresponds for 25*S* (Braca et al., 2004). The closer examination of ^1^H and ^13^C spectra confirmed the aglycone moiety similar to protodiosgenin. The NOESY spectrum showed the correlations between the H-8 and H-18/19 methyl groups which confirmed that the methyl groups were *β*-oriented. The NOESY correlations between H-9/H-14 and H-14/H-16 confirmed them to be *α*-oriented. Further, the ring fusion of B\C and C/D rings was identified as trans, while the D/E ring was confirmed to have cis fusion (Figure 2) by the NOESY correlations (Mimaki et al., 2008). The ^1^H NMR spectrum of compound **2** showed signals characteristic of anomeric protons which were found resonating at *δ*_H_ 4.24 (*d*, *J =*7.8 Hz, Glc C-26), 4.49 (*d*, *J* = 7.84 *Hz*, Glc1-H-1), 4.85 (*br s*, Rha1), and 5.18 (*br s*, Rha 2) with the corresponding anomeric carbon resonances observed at *δ*_C_ 104.67, 100.54, 103.07 and 102.42, respectively, in ^13^C NMR spectrum (Supplementary Figure S11). The sugar component was found to be composed of two units of *D*-glucose and *L*-rhamnose, obtained after acid hydrolysis of compound **2**. It was also confirmed by comparing their ^1^H and ^13^C NMR data and coupling constants with literature reports. The sugar attachment sites were determined by key HMBC correlations (Figure 4). The presence of HMBC correlation between Glc-H-1*′* (δ_H_ 4.49) and C3 (δ_C_ 79.92) of aglycone proved Glc1*′* to be attached at C-3 of the aglycone. There was another key HMBC correlation observed between C-4*′* of Glc1-*′* (*δ*_H_ 4.49) and Rha-H-1*′′* (*δ*_H_ 4.85) which confirmed that Rha1 was located at C-4 of Glc1*′*. Further, the attachment 1*‴* of Rha-2 (*δ*_H_ 5.18) to C-3 of Rha-H-1*′′* (δ_C_ 79.43) was confirmed by correlations found in the HMBC spectrum. There was another correlation between *O*-Glc-H*′′′′* (*δ*_H_ 4.24) and C-26 (*δ*_C_ 76.11). The β -orientation of both *D*-glucopyranosyls was confirmed by their respective relatively large *J* values of anomeric protons (7.8 Hz, *O*-Glc C-26 and 7.8 Hz, *O*-Glc1-H-1). After detailed analysis of this spectral data, the structure of **2** was elucidated as (25*R*)-furost-5-en-3*β*,22*β*,26-triol-3-*O*-*α*-*L*-rhamnopyranosyl-[(1–4)-α-L-rhamnopyranosyl-(1–4)]-*β*-*D*-glucopyranosyl 26-*O*-*β* -D-glucopyranoside (Trilliumoside L).

### 3.2 Biology

#### 3.2.1 Cell growth inhibition studies

According to the previous anti-cancer study reports on *T. govanianum*’s rhizomes, the crude MeOH extract and its polarity-based subfractions have demonstrated different levels of significant cytotoxicity, with IC_50_ values ranging between (5–13 μg/mL) against four human cancer cell lines, namely, urinary bladder (EJ138), breast (MCF7), liver (HepG2), and lung (A549) but there is no individual anti-cancer report on pure isolated compounds of *T. govanianum*. However, there are several anti-cancer studies on steroidal saponins, the major bioactive components of the genus *Trillium* (Yokosuka and Mimaki, 2008b; Hayes et al., 2009) containing mono, di, tri, or tetrasacchride, commonly composed of glucose, rhamnose, apiose, xylose, and arabinose sugar units linked to a β-D-glucosyl moiety at C3 of the aglycone (Gao et al., 2015) (Gao et al., 2015). In one study, Paris saponin VII, a diosgenin-based saponin isolated from *T. tschonoskii Maxim* showed cytotoxicity against MCF7, human colorectal cancer cells-29, and SW-620 (IC_50_ = 9.547, IC_50_ = 1.02 ± 0.05, and 4.90 ± 0.23 μm, respectively). Interestingly, Compound 2 displayed cellular anti-proliferative activity in A-549 cancer cells with IC_50_ values of 1.79 μM with excellent selectivity over fR2 normal cells (Supplementary Table S2). Further, Paris VII induced cell apoptosis in a caspase-3-dependent manner and cell cycle arrest in the G1 Phase (Li et al., 2014). In another study, two new saponins from the underground part of *T. tschonoskii* displayed strong cytotoxic activity against the HepG2 cell line (IC_50_ = 0.499 mmol/L) (Chai et al., 2014). Diosgenin, isolated as a major compound from *Trillium* species, showed a potent cytotoxic effect against HepG2 and HCT116 cells in the MTT assay (Eskander et al., 2013). Based on the above hypothesis and the resemblance of both new compounds (**1** and **2**) with that of previously reported anti-cancer saponins, the current anti-cancer study was designed and demonstrated for the genus *Trillium*.

The anti-cancer potential of crude extracts (CHCl_3,_ 20% aq. MeOH, and one sub-fraction of 20% aq. MeOH) and nine isolated compounds were evaluated for their *in-vitro* cytotoxic potential, which was expressed in percentage of inhibition and IC_50_ values respectively (Supplementary Figures S1, S2) against a panel of human cancer cell lines, namely, lung cancer (A-549, HOP-62), breast (MCF-7, MDA-MB 231), pancreatic (MiaPaCa-2), colon cancer (SW-620, HCT-116), prostate (PC-3) and neuroblastoma (SH-SY5Y) cancer using an SRB assay. The results revealed that among extracts, fraction E.F. (subfraction of 20% aq. MeOH) (IC_50_ µg/mL) exhibited the highest *in-vitro* cytotoxicity, followed by its parent fraction 20%aq. MeOH and CHCl_3_ (IC_50_ µg/mL) as presented in Figure 1. However, among the pure compounds, Compound **1** (IC_50_ µM) and Compound **2** (IC_50_ µM) showed maximum inhibition effects on A-549 and SW-620 cell lines in a concentration-dependent manner (Table 1 S33). Further, three known compounds, diosgenin, protodioscin, and borassoside E, showed good cytotoxic activity on A-549 and SW-620 cell lines. However, compounds TG-01, TG-03, TG-04, TG-05, TG-06, and TG-12 at a concentration of 10 µM did not possess significant cytotoxicity (<45 %) against the tested human cell lines. Compounds **1** and **2** showed the best percentage growth inhibition among all isolated compounds, 75.35 and 92.11 at 10 μM and 50 µM. Out of active compounds **1** and **2**, Compound **2** was further evaluated for detailed *in-vitro* anti-cancer studies by performing DAPI, ROS, mitochondrial membrane potential (MMP), Colony formation assay, and wound healing/scratch assay. However, due to a restricted quantity of Compound **2**, we could not proceed with its *in-vivo* studies.

#### 3.2.2 Compound 2 altered the nuclear morphology assessed by DAPI

**FIGURE 5:**
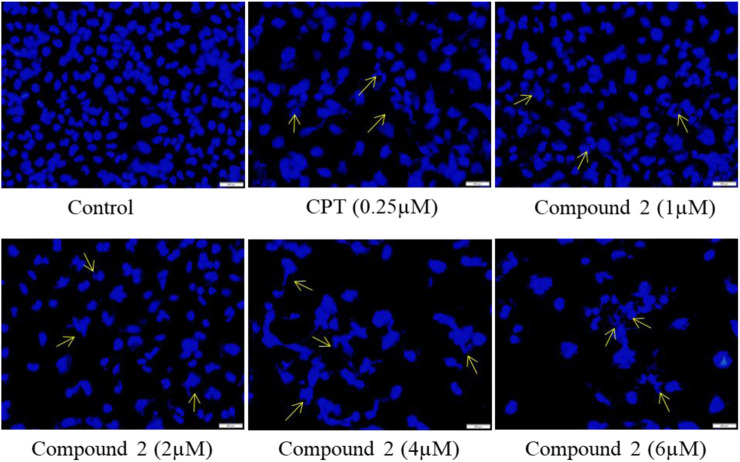
Compound **2** mediated apoptosis in A-549 cells. Cells were cultured for 48 h in the presence of 10% FBS. At 24 h, cells were treated with different concentrations (1, 2, 4, and 6 µM) of compound **2**. Camptothecin was used as a positive control. At 48 h, cells were stained with DAPI (1 μg/mL) dye and monitored by a fluorescence imaging microscope. The yellow arrows indicate apoptotic body formation, nuclear shrinkage, and chromatin condensation.

Cells undergoing apoptosis have characteristic features like apoptotic body formation, nuclear shrinkage, and chromatin condensation (Elmore, 2007). To assess the morphological changes in nuclei, we used DAPI (4′,6-diamidino-2-phenylindole), which is a DNA-specific dye that binds to the minor groove of the A-T region (Biancardi et al., 2013; Pathania et al., 2015). A-549 cells were treated with various concentrations (1, 2, 4, and 6 µM) of Compound **2** for 48 h; Camptothecin (CPT) was used as a positive control. At 48h, cells were washed with PBS, stained with DAPI, and examined under fluorescence microscopy. Higher levels of apoptotic body formation and chromatin condensation were observed in Compound **2** and Camptothecin (0.25 µM) treated A-549 cells in a concentration-dependent manner compared to untreated cells. These morphological changes demonstrate that the treatment of A-549 cells with Compound **2** caused a significant increase in apoptotic body formation in a concentration-dependent manner (Figure 5) and depicts an anti-proliferative effect. These characteristic features were absent in untreated control cells.

#### 3.2.3 Intracellular ROS production induced by compound 2 in A-549 cells

**FIGURE 6:**
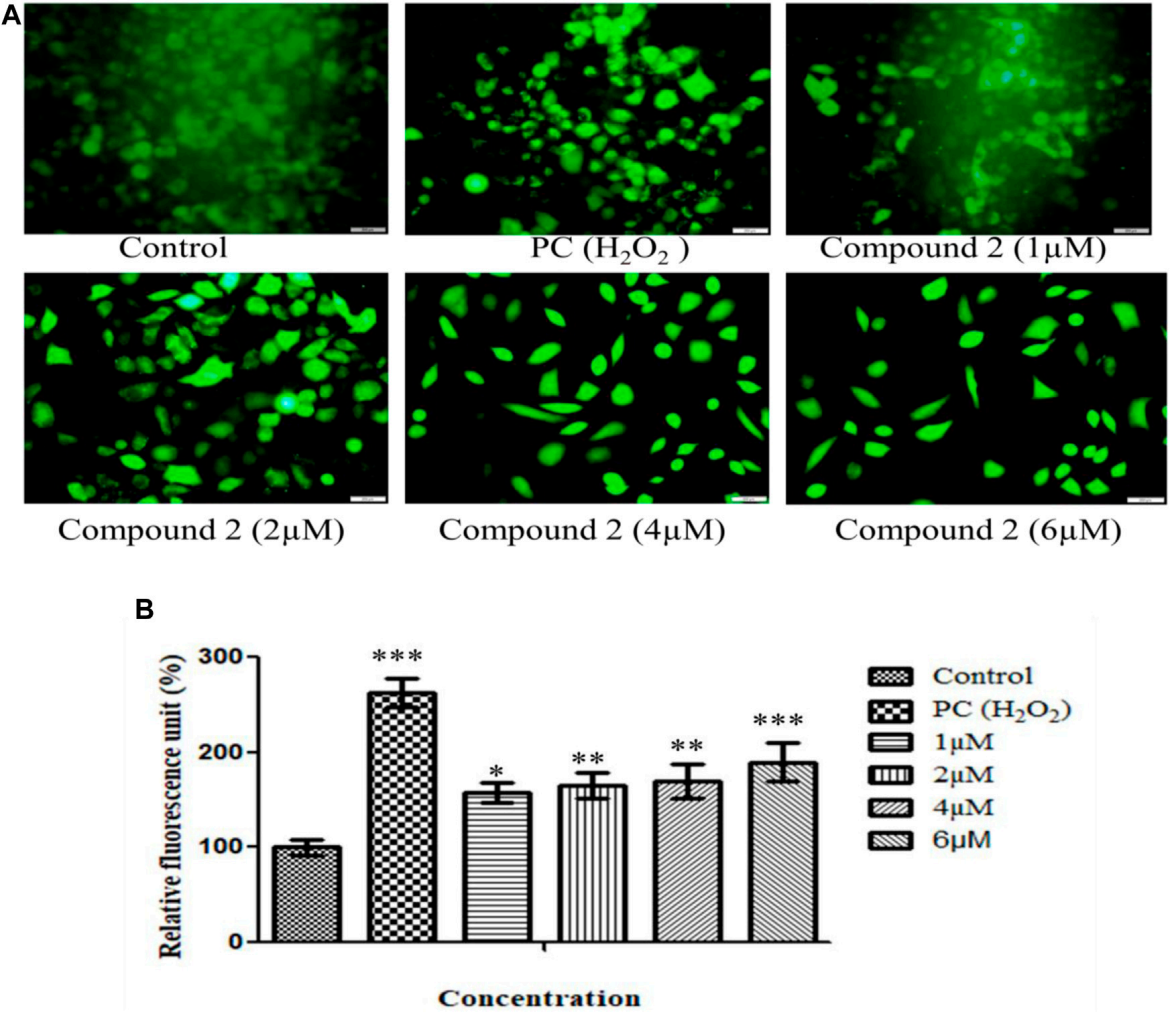
**(A)** Intracellular ROS production induced by compound **2** in A-549 cells: The cells were cultured for 48 h in the presence of 10% FBS. At 24 h, cells were treated with different concentrations (1, 2, 4, and 6 µM) of compound **2**. H_2_O_2_ was taken as a positive control (0.05%). At 48 h, cells were stained with DCFDA dye, and ROS levels were measured using Olympus IX53. Fluorescence at each time point was normalized to untreated control cells; **(B)** Representative histograms showing fluorescence changes in A-549 cells after treatment with various concentrations of compound **2** as determined by the Olympus IX53 microscope. Fluorescent data normalized to positive control showing the average of three experiments. Data are expressed as mean ± S.D. The statistical significance of data was determined by one-way analysis of variance (ANOVA) (*p < 0.05, **p < 0.01, and ***p < 0.001).

ROS are pivotal in many biological processes. ROS are a hallmark of apoptosis. Increased intracellular ROS levels damage the membrane lipids, intra-cellular proteins, organelles, and nucleic acids, which trigger cell cycle arrest and apoptosis in cancer cells (Perillo et al., 2020). Therefore, to examine the impact of Compound **2** on ROS production, A549 cells were treated with various concentrations of compound **2** (1, 2, 4, and 6 µM), and intracellular ROS levels were analyzed using a fluorescent probe DCFH-DA. The fluorescence intensity is a quantitative estimation of ROS generation in cancer cells. A significant increase in intracellular ROS levels was observed in Compound-treated A-549 cells compared to untreated cells. Higher levels of ROS were observed in the positive control (H_2_O_2_-treated cells). This study shows that compound **2** induces ROS generation in A-549 cells in a concentration-dependent manner. Green signal cells were quantified using ImageJ software and presented in the histogram (Figures 6A, B).

#### 3.2.4 Compound 2 lowers mitochondrial membrane potential

**FIGURE 7:**
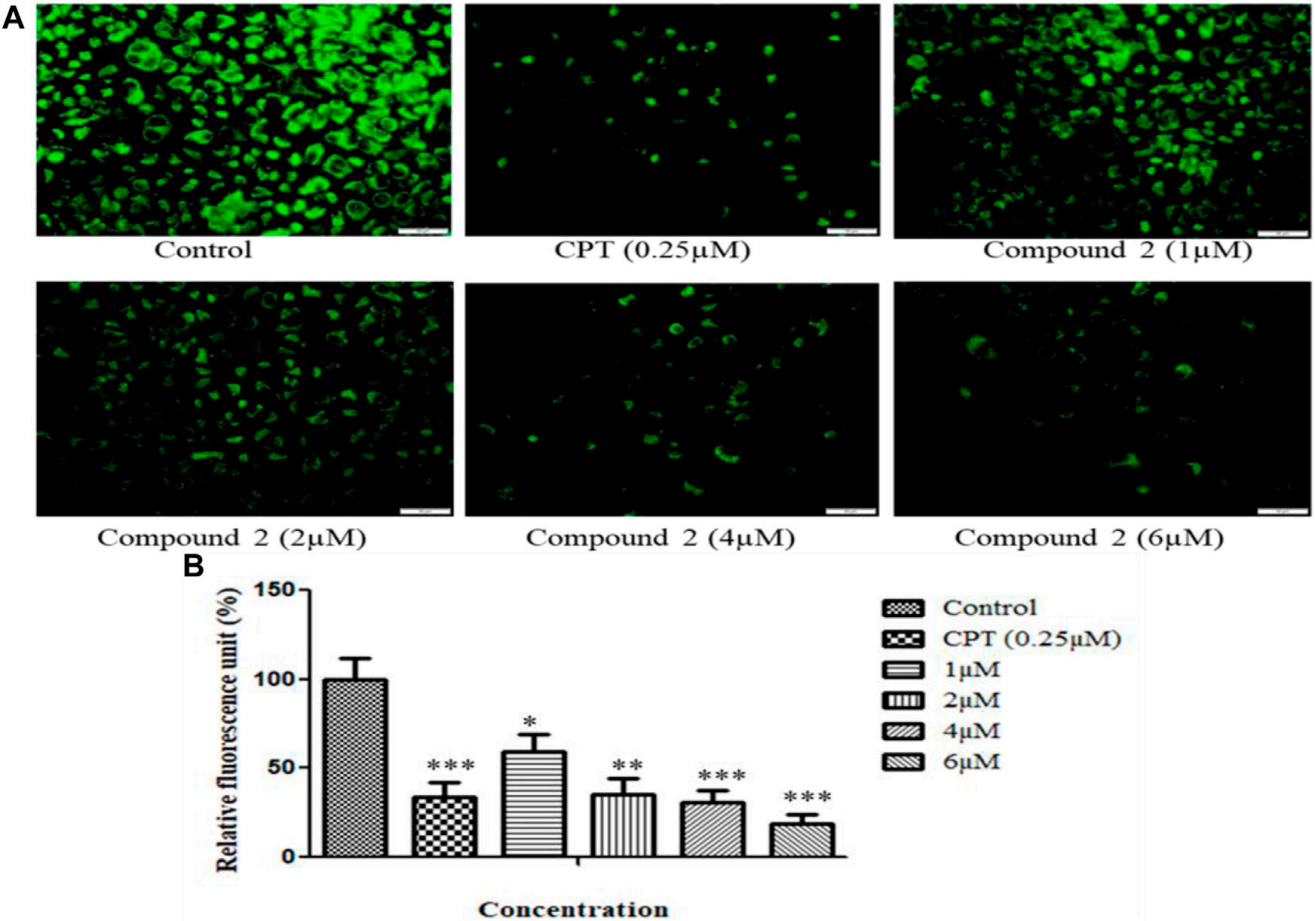
**(A)** Loss of mitochondrial membrane permeability induced by compound **2** in A-549 cells. The cells were cultured for 48 h in the presence of 10% FBS. At 24 h, cells were treated with different concentrations (1, 2, 4, and 6 µM) of compound **2**. Camptothecin was used as a positive control. At 48 h, cells were stained with Rhodamine-123, and loss of MMP was quantified using an Olympus IX53 microscope. Fluorescence at each time point was normalized to untreated control cells. **(B)** Representative histograms showed fluorescence changes in A-549 cells after treatment with various concentrations of compound **2** as determined by the Olympus IX53 microscope. Fluorescent data were normalized to the positive control, showing the average of three experiments. Data are expressed as mean ± S.D. The statistical significance of data was determined by one-way analysis of variance (ANOVA) (*p < 0.05, **p < 0.01, and ***p < 0.001).

The loss of mitochondrial membrane permeability and release of cytoplasmic apoptogenic stimuli leads to MMP loss and eventually causes cell death (Gottlieb et al., 2003). A fluorescent dye, rhodamine-123, was used to analyze the change in MMP. An Olympus IX53 imaging microscope monitored the quenching of fluorescence. The loss of mitochondrial membrane integrity is directly related to the fluorescence decay rate. The mitochondrial membrane destabilization causes leakage of rhodamine-123, which in turn lowers the fluorescence intensity (Kumar et al., 2016). After treatment with various concentrations (1, 2, 4, and 6 µM) of compound **2** and camptothecin (0.25 µM), A-549 cells showed a significant reduction in MMP. Camptothecin was used as a positive control. Compound **2** induces the depolarization of mitochondrial membrane potential (low ∆Ψmt) in a concentration-dependent manner. Green signal cells were quantified using ImageJ software and presented in the histogram (Figures 7A, B).

#### 3.2.5 Compound 2 inhibited *in vitro* cell migration during wound healing assay in A-549 cells

**FIGURE 8:**
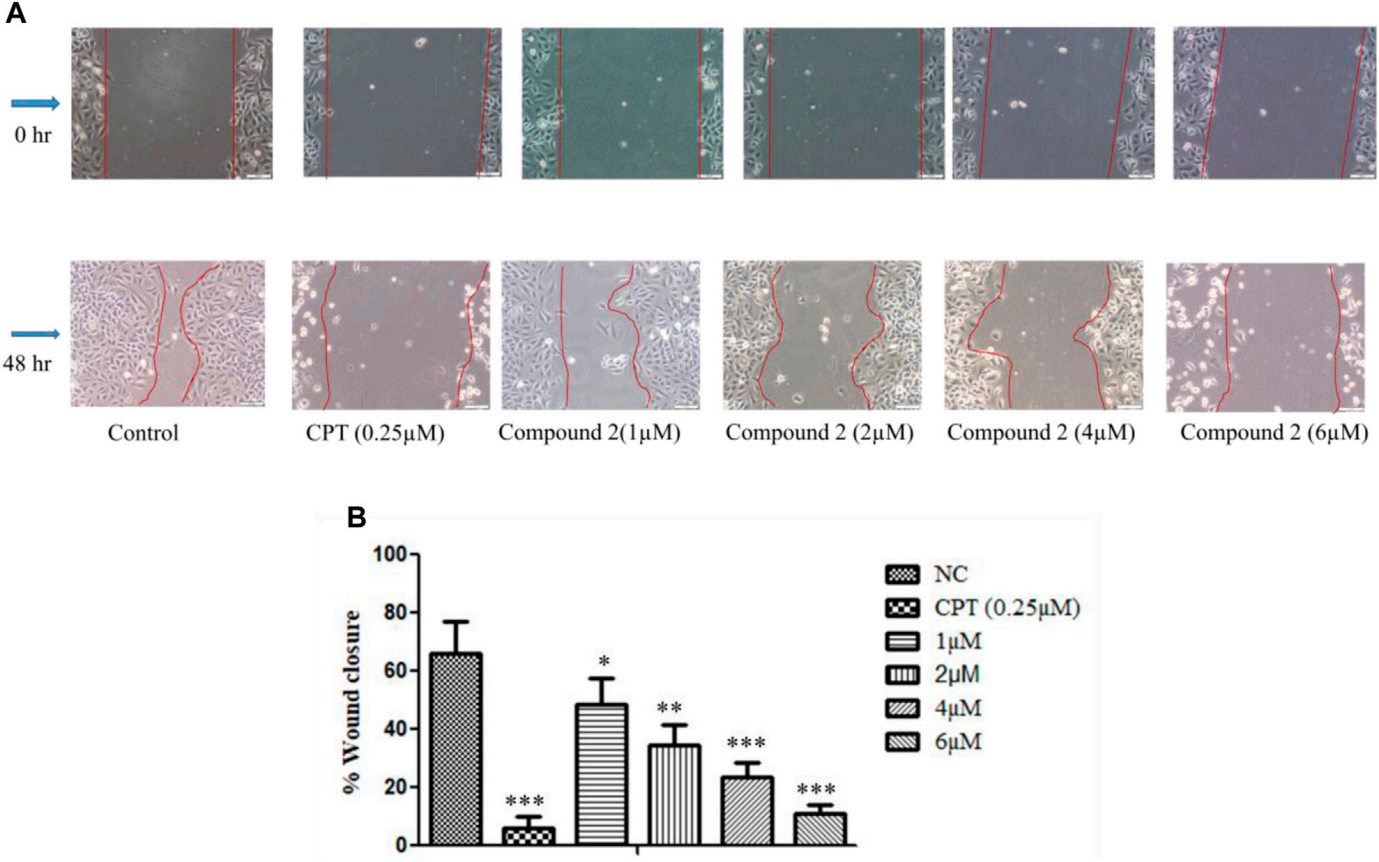
*In vitro* wound healing assay. **(A)** A-549 cells treated with Compound **2** for 48 h reduced the migration of the cell as the concentration of the compound was increased. **(B)** Wound closure area with control using ImageJ software. Data are expressed as mean ± S.D. The statistical significance of data was determined by one-way analysis of variance (ANOVA) (*p < 0.05, **p < 0.01, and ***p < 0.001).

In this experiment, A-549 cell monolayers were scratched and treated with different concentrations of Compound **2**, i.e., 1, 2, 4, and 6 µM for 48 h. Images were taken at 0 and 48 h to quantitatively assess the percentage reduction in cell migration (Rodriguez et al., 2005). It was observed that inhibition of cell migration took place in a concentration-dependent manner as compared to the control. This indicates the potential of Compound **2** to retard cancer cell growth (Figure 8).

#### 3.2.6 Compound 2 inhibited cell proliferation during colony formation assay in lung cancer cells (A-549)

**FIGURE 9:**
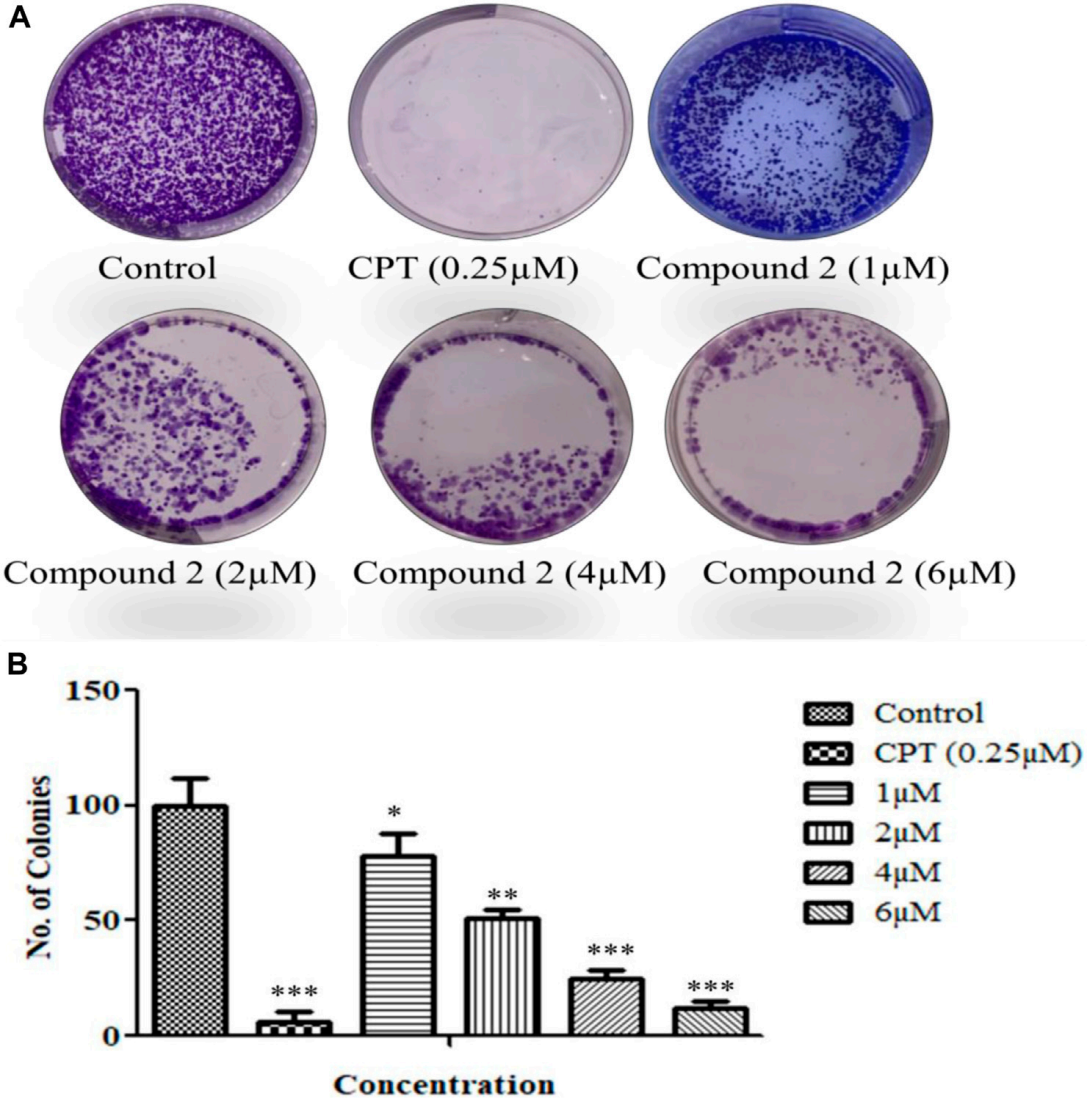
**(A)** A colony formation assay was performed over A-549 cells. **(B)** The number of colonies was counted and plotted as a bar graph. Camptothecin (0.25 µM) was used as a positive control. Data are expressed as mean ± S.D. The statistical significance of data was determined by one-way analysis of variance (ANOVA) (*p < 0.05, **p < 0.01, and ***p < 0.001).

The cells’ proliferation capabilities (the potential of a single cell to grow into a colony) were measured by an *in vitro* clonogenic assay (Munshi et al., 2005). A-549 cells were seeded in a 6-well flat transparent well plate and treated with 1, 2, 4, and 6 µM concentrations of Compound **2**. Compound **2** significantly inhibited colony formation in A-549 cells as the concentration increased along with the inhibition effect (Figure 9).

#### 3.2.7 Western blotting

**FIGURE 10:**
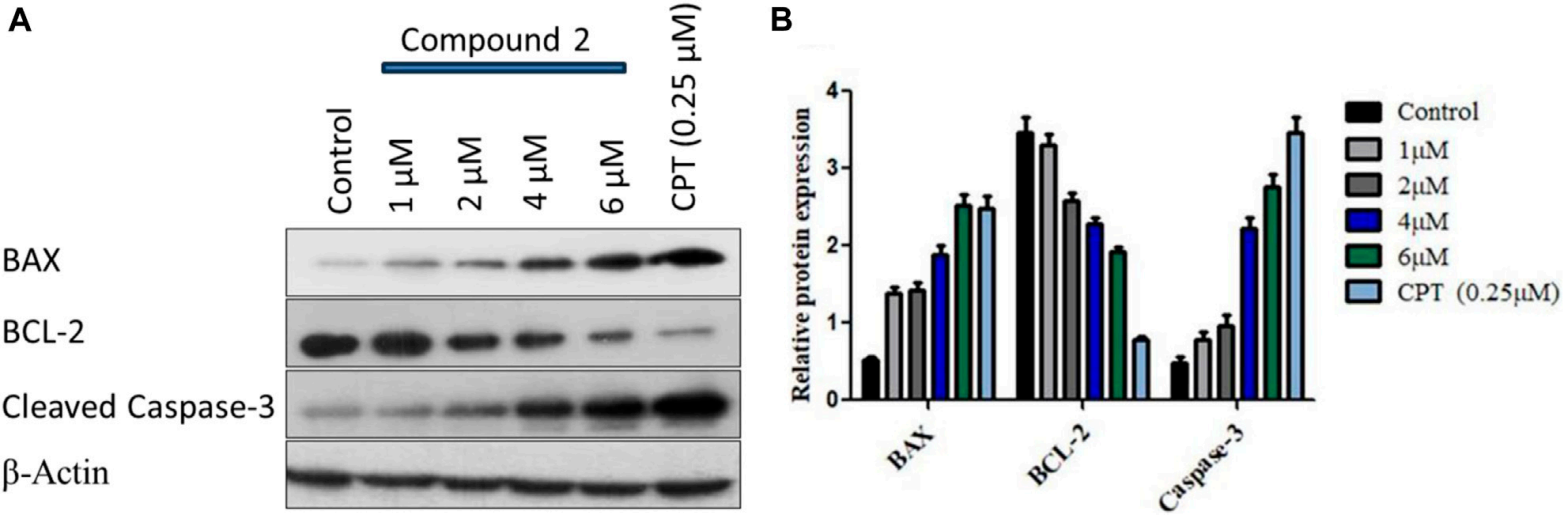
**(A)** Immunoblot depiction of BAX, BCL-2, and cleaved caspase-3 in A-549 cells by Compound 2 for 48 h **(B)** represents the densitometric Western blot analysis. Data were calculated from 3 independent experiments with *p* < 0.05.

We observed a dose-dependent reduction in mitochondrial membrane potential after treatment with Compound **2**, accompanied by a significant elevation in intracellular ROS levels in A-549 cells compared to untreated cells. Furthermore, we noted higher levels of apoptotic body formation and chromatin condensation in treated A-549 cells in a concentration-dependent manner relative to untreated cells. These morphological alterations signify that the treatment of A-549 cells with Compound **2** induces a substantial increase in apoptotic body formation in a concentration-dependent manner, underscoring its anti-proliferative effect. For the analysis of programmed cell death (apoptosis) induction by Compound **2** in A-549 cells, Western blotting was conducted with BCL-2, BAX, and cleaved caspase-3. BCL-2 serves as an anti-apoptotic marker, while BAX and cleaved caspase-3 are pro-apoptotic. We observed a dose-dependent increase in the expression of BAX and a corresponding decrease in the expression of BCL-2 in response to Compound **2** in the A549 cell line. The typical Western blot images of BAX and BCL-2 are presented in Figure 10.

Subsequently, the activation of caspase-3 was assessed by monitoring procaspase-3 cleavage through Western blotting. The blot, displayed in Figure 10 (3rd panel from the upper side), illustrates a clear augmentation in procaspase-3 cleavage with increasing doses of Compound 2. The expression levels of BCL-2, BAX, and procaspase-3 were quantified for each dose using ImageJ software, and the relative expressions, along with standard deviations, were calculated and plotted in Figure 10B using GraphPad Prism. β-Actin served as the loading control. This data suggests that Compound **2** induces the activation of caspase-3 after the cleavage of pro-caspase-3, establishing a dose-dependent activation of caspase-3 in A-549 cells.

## 4 Conclusion

Over the past years, *T. govanianum* has gained the attention of researchers because of very little exploration of its phytochemistry and pharmacological studies, especially in cancer research. In the present study, we isolated and characterized two new steroidal saponins from the rhizomes of *T. govanianum.* Compounds **1** and **2** exhibited significant cytotoxic activity against human lung and colon cancer cell lines in a considerable micromolar range. Trilliumoside K (**1**) showed significant cytotoxicity with IC_50_ values of 1.83 and 1.85 µM on A-549 (Lung) and SW-620 (Colon) cell lines, whereas the IC_50_ value against the A-549 cell line of trilliumoside L (**2**) was found to be 1.79 µM. Mechanistic anti-cancer assays were performed on Compound **2**, revealing noteworthy changes such as MMP reduction, increase in ROS production, inhibition of anti-apoptotic protein BCL-2, and activation of BAX and caspase-3. Compound **2** showed cellular anti-proliferative activity in A-549 cancer cells. A-549 is identified as a sensitive cell line in *in vitro* cytotoxicity assays, with a safety ratio of ∼6 (IC_50_ against fR_2_ normal cell line versus IC_50_ in A549 cancer cell line). All these changes were responsible for the induction of apoptosis in the A-549 cancer cell line, laying the foundation of Compound **2** as a lead molecule in anti-cancer drug discovery.

## Data Availability

The datasets presented in this study can be found in online repositories. The names of the repository/repositories and accession number(s) can be found in the article/Supplementary Material.
