# Supplementary material for: Trilliumosides K and L, two novel steroidal saponins from rhizomes of Trillium govanianum, as potent anti-cancer agents targeting apoptosis in the A-549 cancer cell line
Source: Front Chem. 2023 Dec 22;11:1306271. doi: 10.3389/fchem.2023.1306271 (PMC10767554; doi:10.3389/fchem.2023.1306271)
Supplement: Supplementary file 1 [file DataSheet1.pdf]

Trilliumosides K-L, two novel steroidal saponins from rhizomes of *Trillium govanianum* as potent anticancer agents targeting apoptosis in A-549 cancer cell line.

Bashir Ahmad Lone[1,2†], Misbah Tabassum[1,3†], Anil Bhushan[1,2], Dixhya Rani[1,2], Urvashi Dhiman[1,2], Ajaz Ahmad[4], Hilal Ahmad Mir[5], Prem N. Gupta[1,3], D. M. Mondhe[1,3], Sumeet Gairola[1,6] and Prasoon Gupta[1,2*]

[1]CSIR-Human Resource Development Centre, Academy of Scientific and Innovative Research, Ghaziabad, India, [2]Natural Products and Medicinal Chemistry Division, CSIR-Indian Institute of Integrative Medicine, Jammu, India, [3]Pharmacology Division, CSIR-Indian Institute of Integrative Medicine, Jammu, India, [4]Department of Clinical Pharmacy, College of Pharmacy, King Saud University, Riyadh 11451, Saudi Arabia, [5]Department of Ophthalmology, Pathology, and Cell Biology, Columbia University, New York, NY, United States, [6]Plant Science and Agrotechnology Division, CSIR-Indian Institute of Integrative Medicine, Jammu, India

Abstract

Two novel steroidal saponins, trilliumosides K (**1**) and L (**2**) were isolated from the rhizomes of *Trillium govanianum* led by bioactivity-guided phytochemical investigation along with seven known compounds govanoside D (**3**) protodioscin (**4**), borassoside E (**5**), 20-hydroxyecdysone (**6**), 5,20-hydroxyecdysone (**7**), govanic acid (**8**) and diosgenin (**9**). The structure of novel compounds 1-2 were established using analysis of spectroscopic data including 1D, 2D NMR and HR-ESI-MS data. All isolated compounds were evaluated for in-vitro cytotoxic activity against a panel of human cancer cell lines. Compound (**1**) showed significant cytotoxic activity against A-549 (Lung) and SW-620 (Colon) cancer cell lines with IC50 values of 1.83 & 1.85 µM, whereas compound (**2**) IC50 value against A-549 cell line was found to be 1.79 µM respectively. Among previously known compounds (**3**), (**5**) and (**9**) their cytotoxic IC50 value was found to be in the range of 5-10 µM. Comprehensive anticancer investigation revealed that compound (**2**) inhibited in-vitro migration and colony forming capability in the A-549 cell line. Additionally, the mechanistic analysis of compound (**2**) on the A-549 cell line indicated distinctive alterations in nuclear morphology, increased reactive oxygen species (ROS) production, and decreased levels of mitochondrial membrane potential (MMP). By upregulating the pro-apoptotic protein BAX and downregulating the anti-apoptotic protein BCL-2, the aforementioned actions eventually cause apoptosis, a

crucial hallmark in cancer research, which activates Caspase-3. To the best of our knowledge, this study reports the first mechanistic anticancer evaluation of the compounds isolated from the rhizomes of *Trillium govani*anum with remarkable cytotoxic activity in the desired micromolar range.

Keywords: *Trillium govanianum,* Saponins, Steroidal glycosides, A-549, BAX, BCL-2, cytotoxicity.

Figure S1. ^{1}HNMR spectrum of compound **1 (TG-07 B3)** (MeOD$_{6}$, 500 MHz)

Figure S2.^{13}C NMR spectrum of compound **1** (MeOD$_{6}$, 100 MHz)

Figure S3. DEPT spectrum of compound **1** (MeOD$_{6}$, 100 MHz)

Figure S4. HSQC spectrum of compound **1**

Figure S5. HMBC spectrum of compound **1**

Figure S6. COSY spectrum of compound **1**

Figure S7. NOESY spectrum of compound **1**

Figure S8. ESI-MS spectrum of compound **1**

Figure S9. GC/MS analysis of compound **1**

Figure S10. ^{1}HNMR spectrum of compound **2 (TG-09)** (MeOD$_{6}$, 500 MHz)

Figure S11. ^{13}C NMR spectrum of compound **2** (CD_3OD, 500 MHz)

Figure S12. DEPT spectrum of compound **2** (CD_3OD_{6}, 500 MHz)

Figure S13. HSQC spectrum of compound **2**

Figure S14. HMBC spectrum of compound **2**

Figure S15. COSY spectrum of compound **2**

Figure S16. NOESY spectrum of compound **2**

Figure S17. HR-ESIMS spectrum of compound **2**

Figure S18. GC/MS analysis of compound **2**

Figure S19. ^{1}HNMR spectrum of compound **3 (TG-12)** (CD_3OD, 500 MHz)

Figure S20.^{13}C NMR spectrum of compound **3** (CD_3OD, 100 MHz)

Figure S21. ^{1}HNMR spectrum of compound **4 (TG-04)** (CD_3OD, 500 MHz)

Figure S22.^{13}C NMR spectrum of compound **4** (CD_3OD, 100 MHz)

Figure S23. ^{1}HNMR spectrum of compound **5 (TG-08)** (CD_3OD, 500 MHz)

Figure S24. ^{13}C NMR spectrum of compound **5** (CD_3OD, 100 MHz)

Figure S25. ^{1}HNMR spectrum of compound **6 (TG-01)** (CD_3OD, 500 MHz)

Figure S26. ^{13}C NMR spectrum of compound **6** (CD_3OD, 100 MHz)

Figure S27. ^{1}HNMR spectrum of compound **7 (TG-02)** ($CDCl_3$, 500 MHz)

Figure S28. ^{13}C NMR spectrum of compound **7** ($CDCl_3$, 100 MHz)

Figure S29. ^{1}HNMR spectrum of compound **8 (TG-05)** (CD_3OD, 500 MHz)

Figure S30. ^{13}C NMR spectrum of compound **8** ($MeOD_6$, 100 MHz)

Figure S31. ^{1}HNMR spectrum of compound **9 (TG-06)** (CD_3OD, 500 MHz)

Figure S32. ^{13}C NMR spectrum of compound **9** (CD_3OD, 100 MHz)

Table 1 S33. Growth inhibitory effect of Extracts and enriched fractions

Table 2 S34. SRB assay-based screening results.

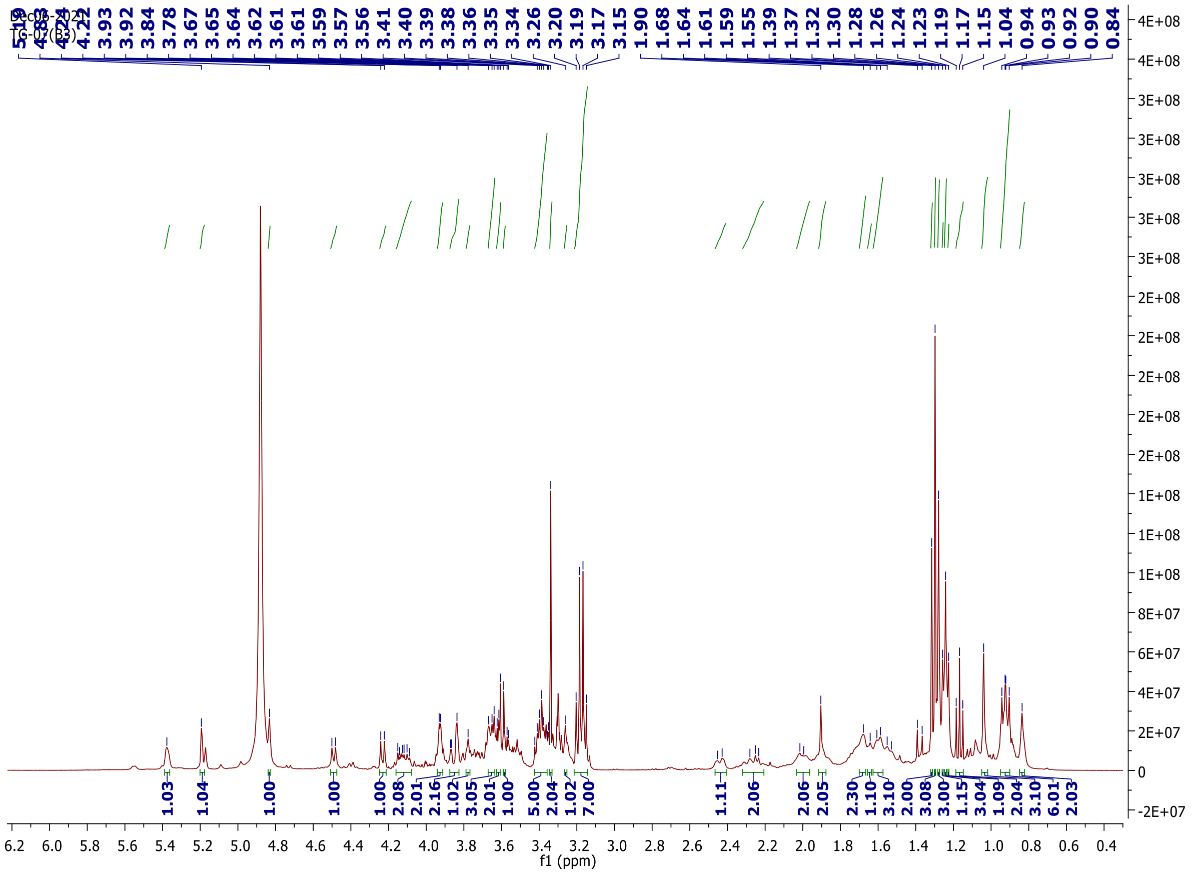

Figure S1. ^{1}H NMR (400 MHz, CD_3OD) of compound TG 07 B3

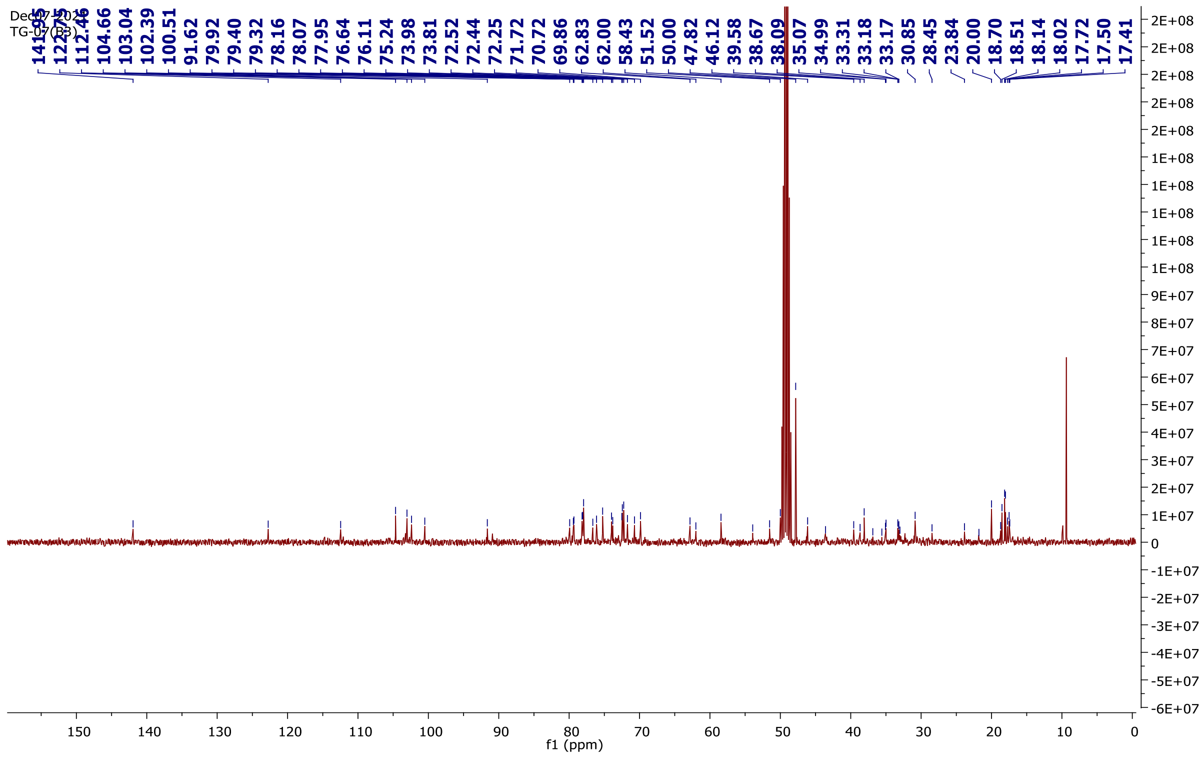

Figure S2. ^{13}C NMR (400 MHz, CD_3OD) of compound TG 07 B3

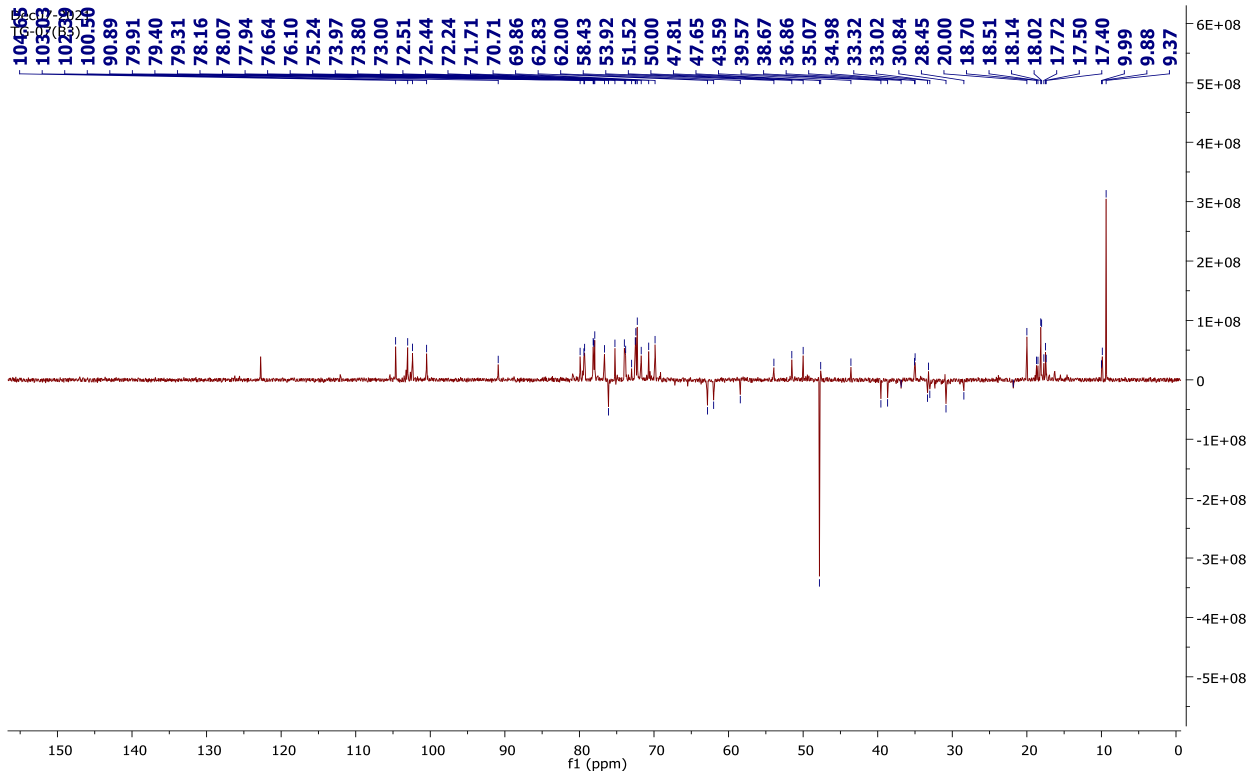

Figure S3. DEPT Spectra of compound TG 07 B3

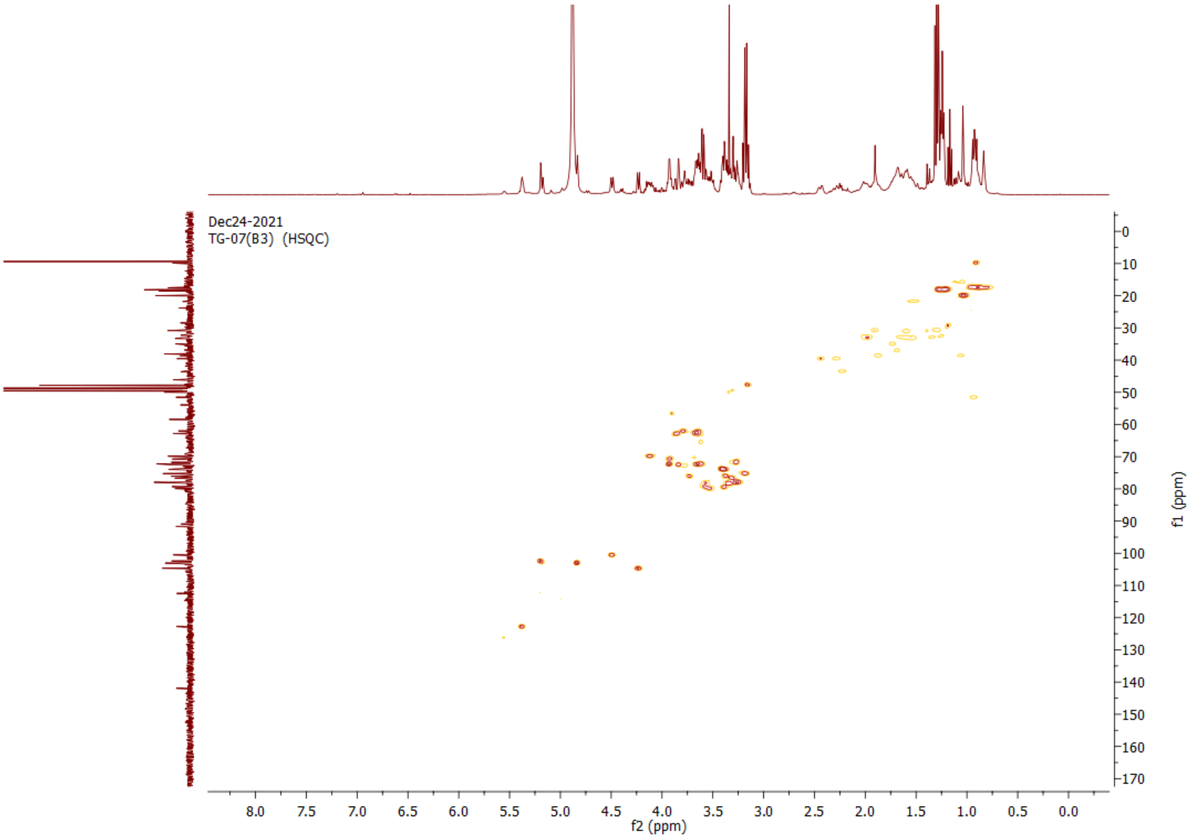

Figure S4. HSQC Spectra of compound TG 07 B3

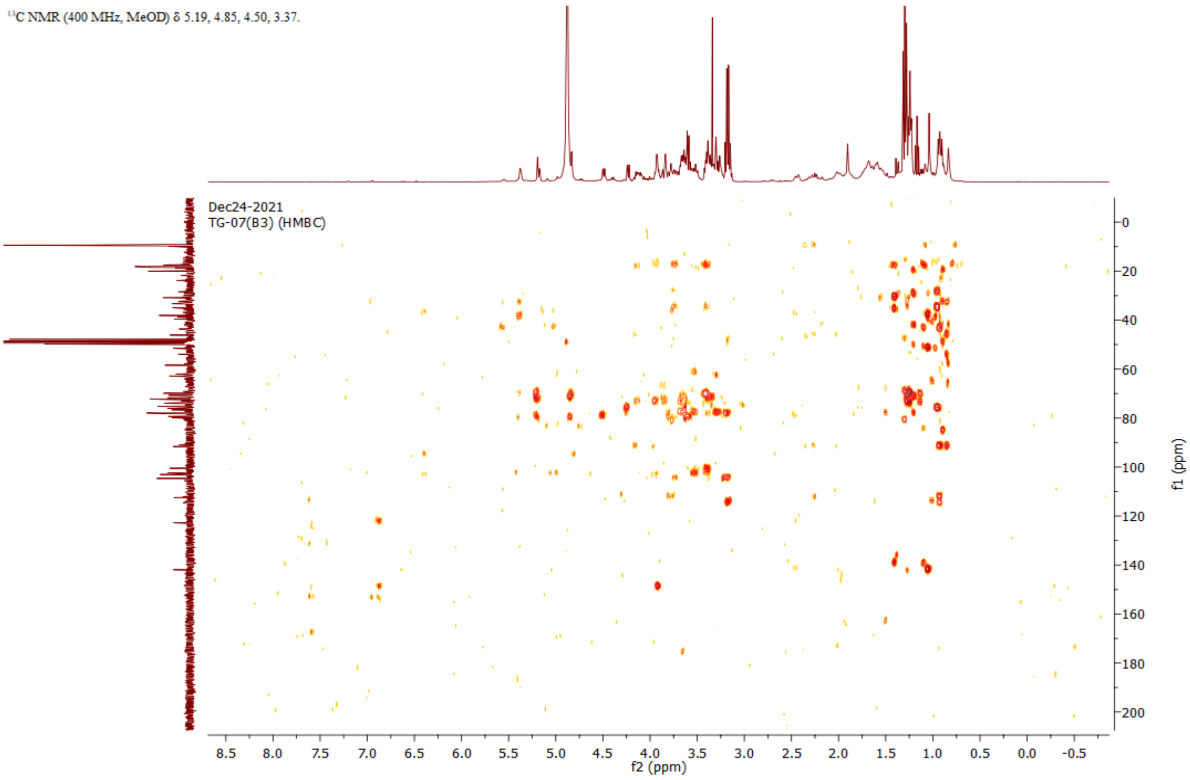

Figure S5. HMBC Spectra of compound TG 07 B3

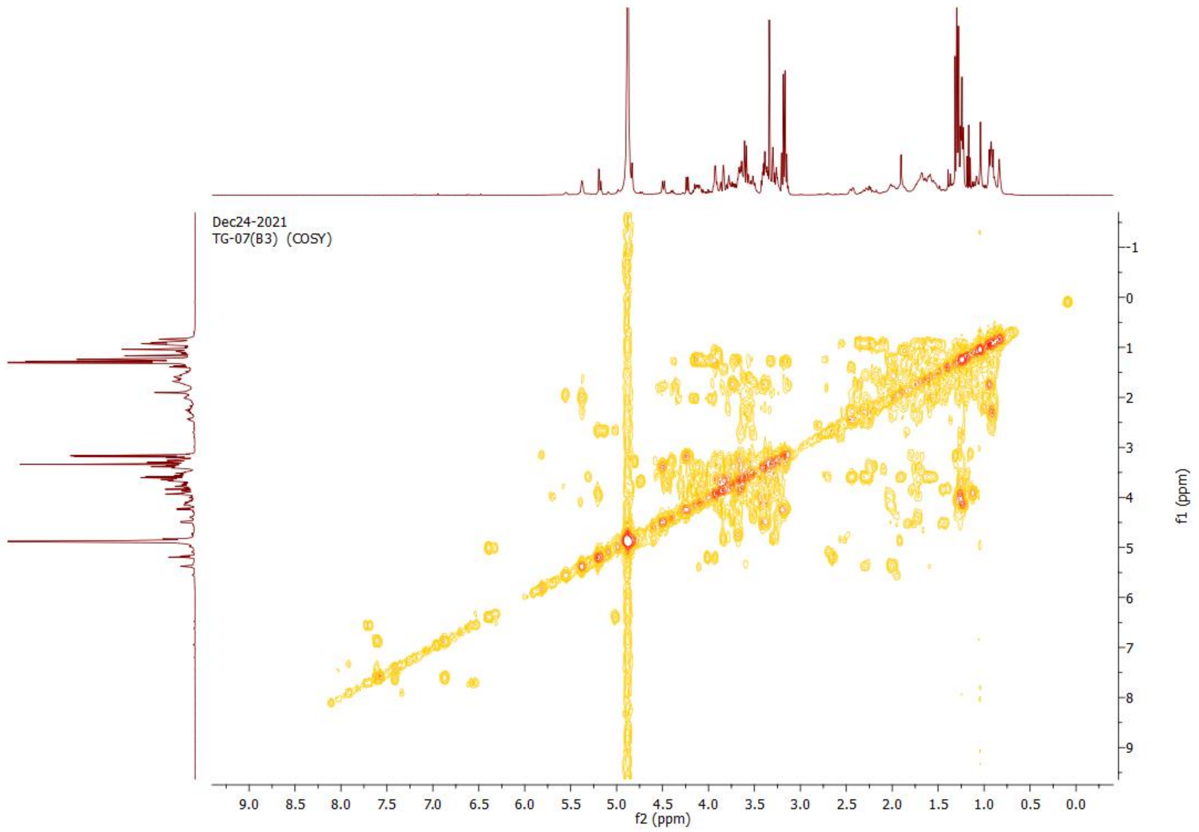

Figure S6. COSY Spectra of compound TG 07 B3

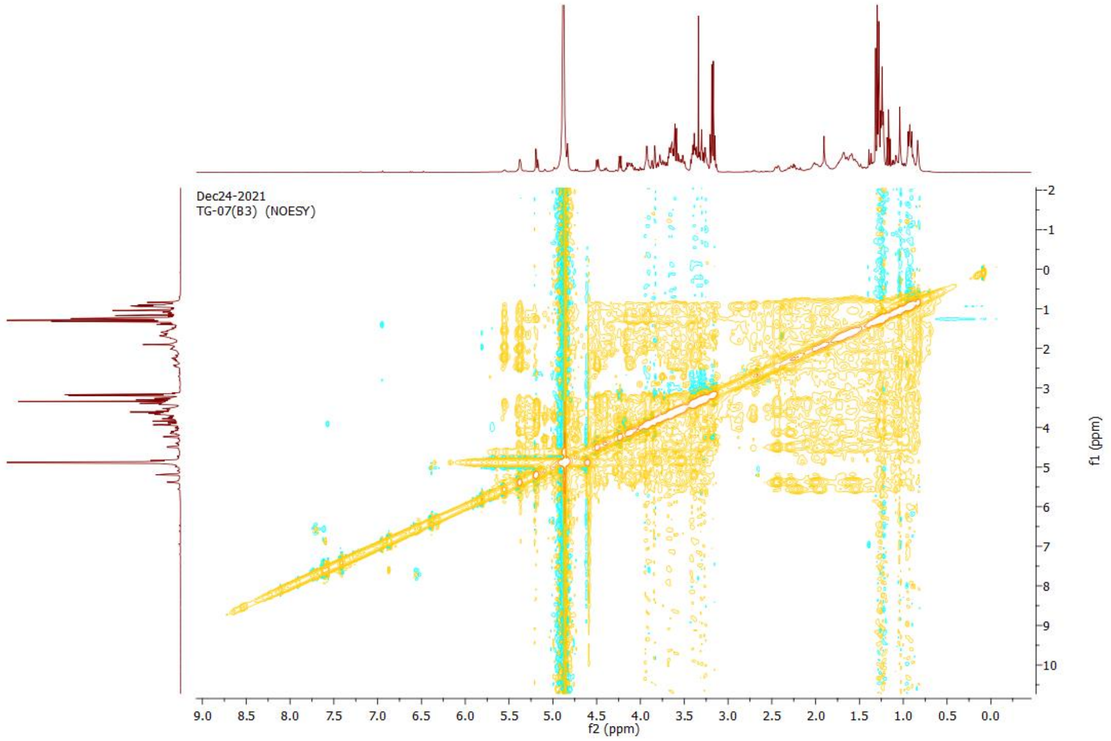

Figure S7. NOESY Spectra of compound TG 07 B3

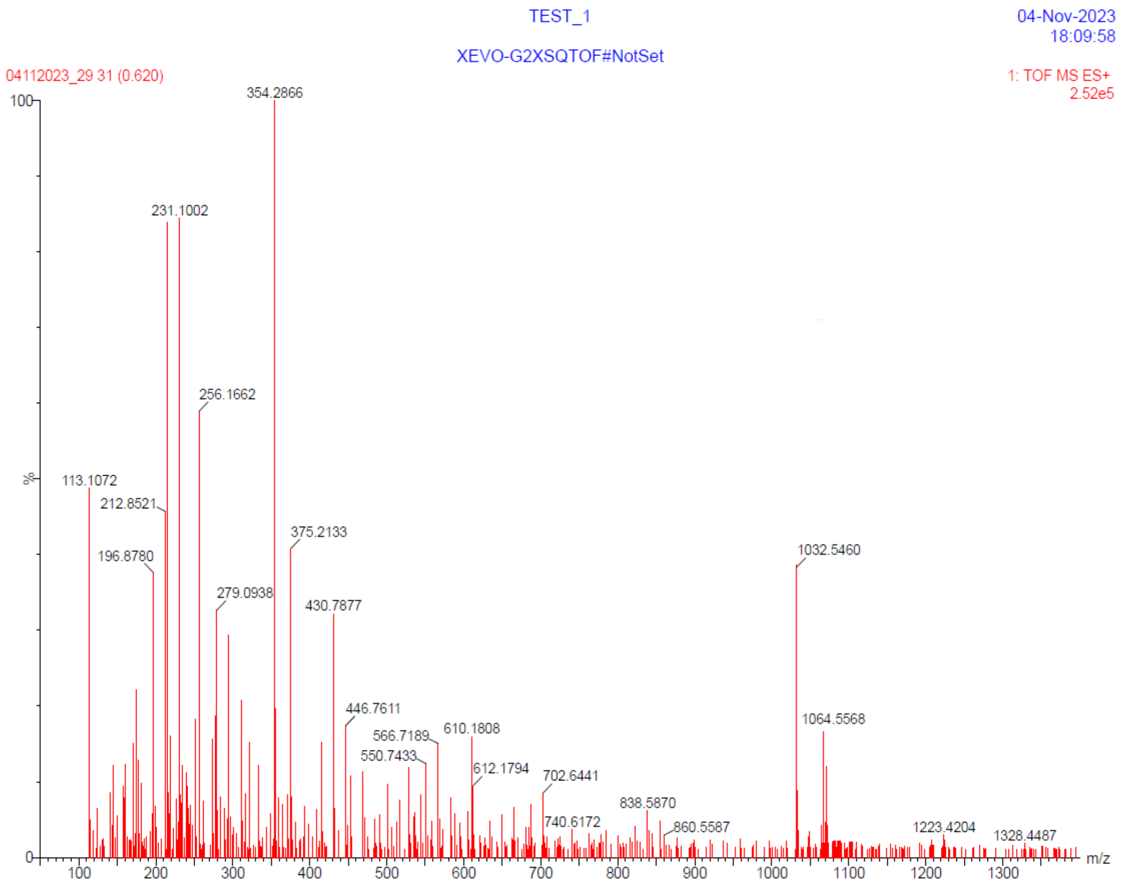

Figure S8A. LC/MS of Compound 1

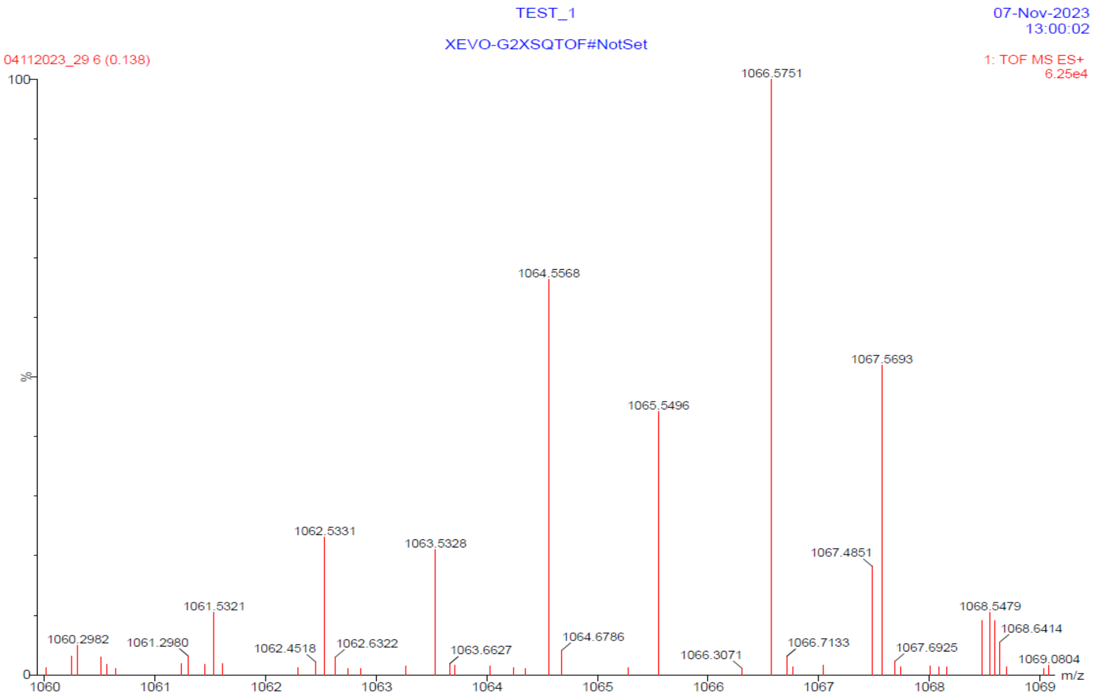

Figure S8B. LC/MS of Compound 1.

IIIM GCMS ANALYSIS REPORT

Sample Information

Analyzed by	: ADMIN
Analyzed	: 1/27/2022 12:05:44 AM
Sample Type	: Unknown
Sample Name	: TG+09
Sample Amount	: 1
Vial #	: 4
Injection Volume	: 0.50
Data File	: D:\GCMS DATA\LIQUID\JAN 2022\25.01.2022\25.01.2022 BHARTI035.qgd
Method File	: D:\GCMS METHOD\GCMS-GENERAL AMIT 1.qgm
Tuning File	: D:\Tuning\Tuning-NM-F1 18.01.2022.qgt
Modified by	: Admin
Modified	: 1/27/2022 12:50:36 AM

Chromatogram TG+09 D:\GCMS DATA\LIQUID\JAN 2022\25.01.2022\25.01.2022 BHARTI035.qgd

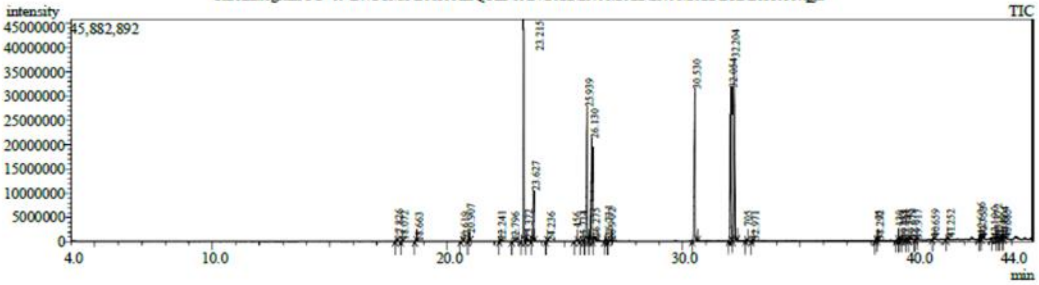

Peak Report TIC

Peak#	R.Time	Area	Area%	Height	Similarity	Name
1	23.215	111246953	6.60	45790083	93	Acetyl 2,3,4-tri-O-acetyl-6-deoxy-.alpha.-D-glucopyranosid
2	23.627	26047880	1.89	10498744	96	.alpha.-l-Talopyranoside, methyl 6-deoxy-, triacetate
3	25.939	99084960	14.79	27692680	95	Acetyl 2,3,4-tri-O-acetyl-6-deoxy-.alpha.-D-glucopyranoside
4	26.130	73864469	11.02	21246385	92	115.05 Tetraacetyl .beta.-l-rhamnose
5	30.530	113234727	16.90	31488565	97	Methyl 2,3,4,6-tetra-O-acetyl-.beta.-D-glucopyranoside
6	32.054	915066032	23.66	31798909	94	beta.-D-Galactopyranose, pentaacetate
7	32.204	110910512	16.55	37520918	93	beta.-D-Glucopyranose pentaacetate

Figure S9. GC/MS analysis of compound 1 (TG 07 B3)

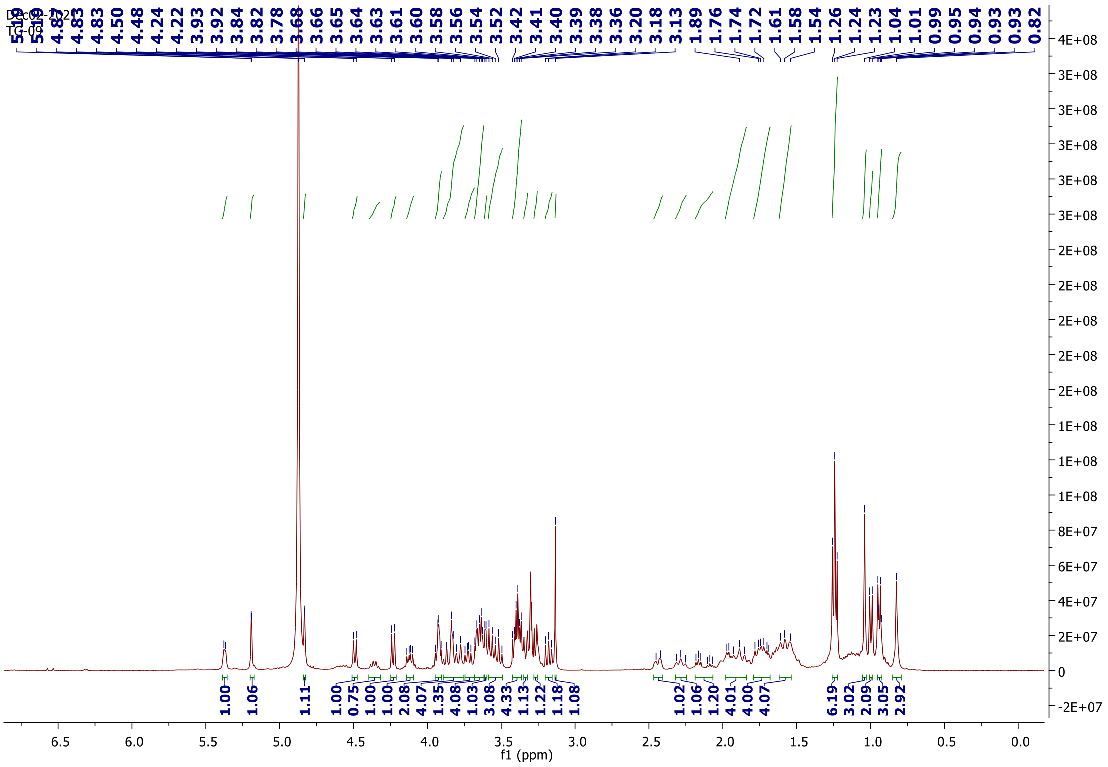

Figure S10. ^{1}H NMR (400 MHz, CD_3OD) of compound TG 09

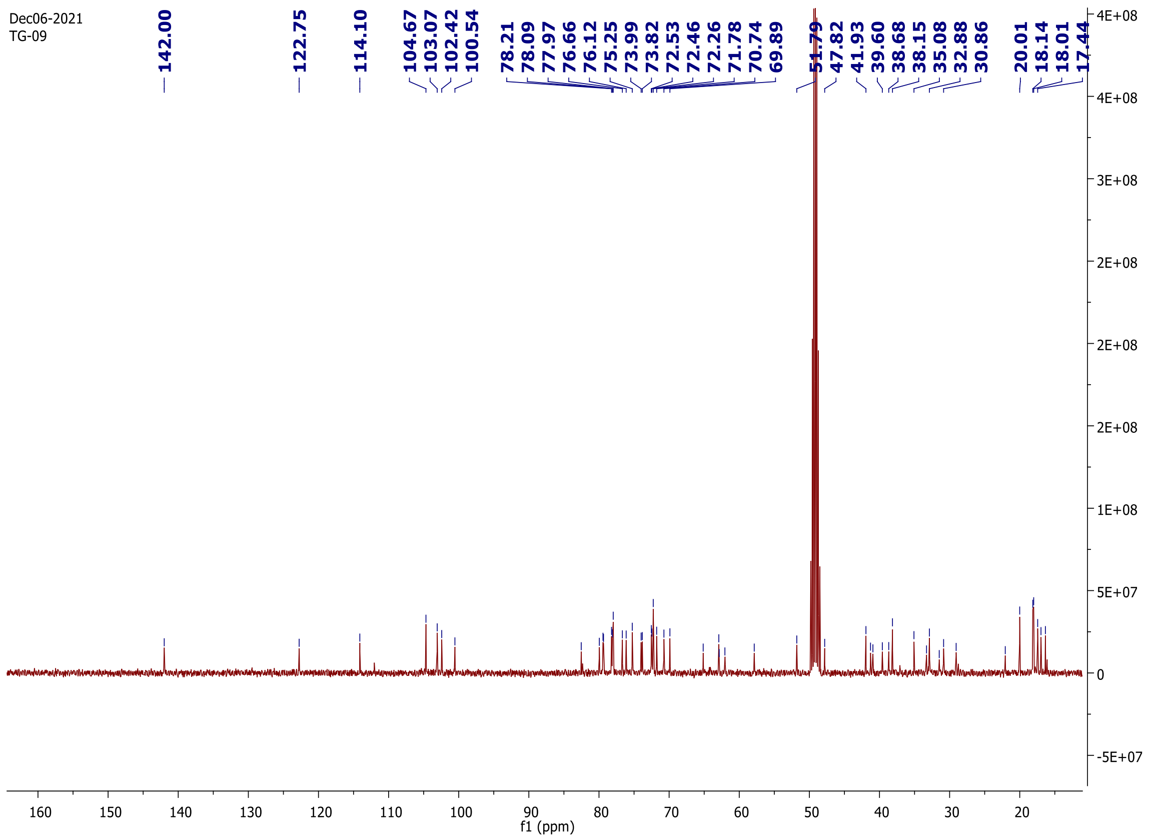

Figure S11. ^{13}C NMR (400 MHz, CD_3OD) of compound TG 09

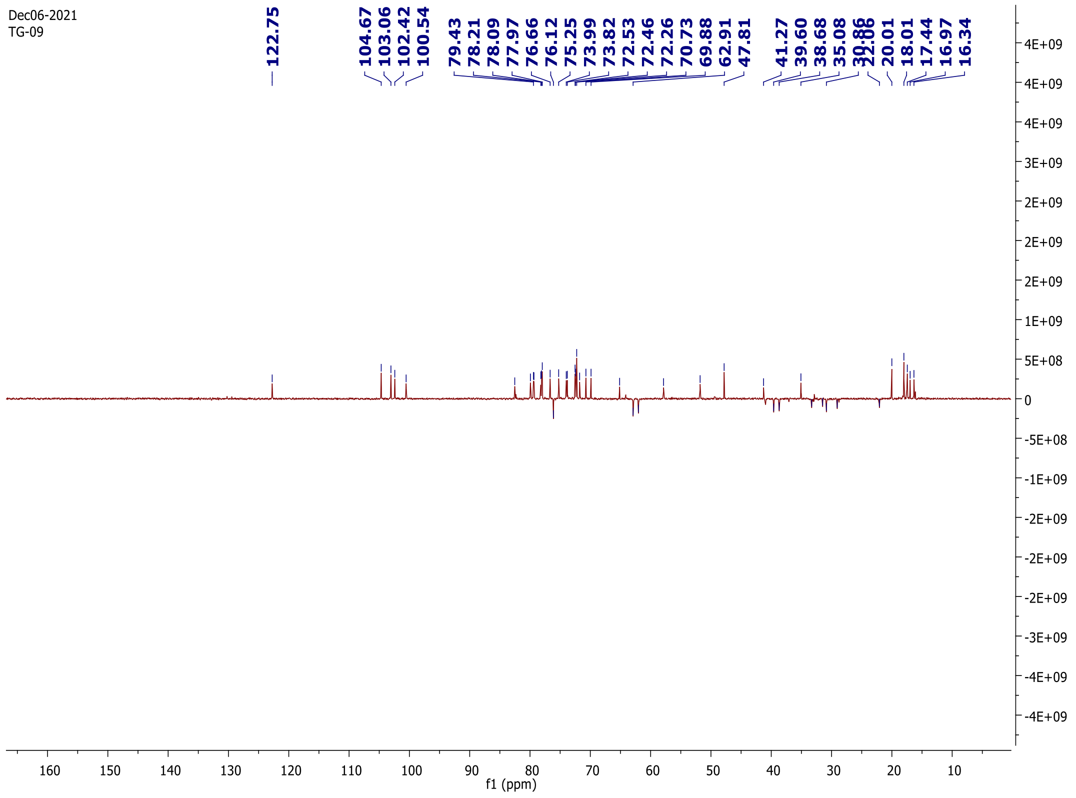

Figure S12. DEPT Spectra of compound TG 09

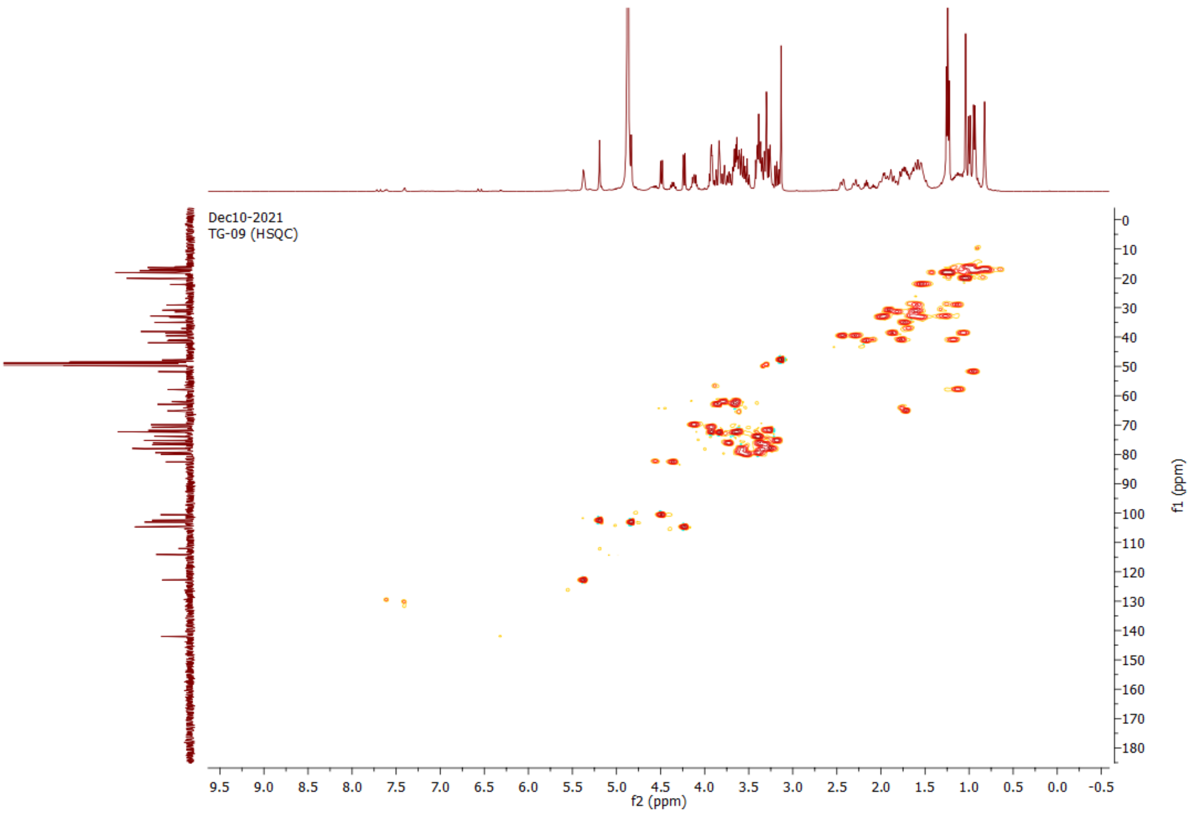

Figure S13. HSQC Spectra of compound TG 09

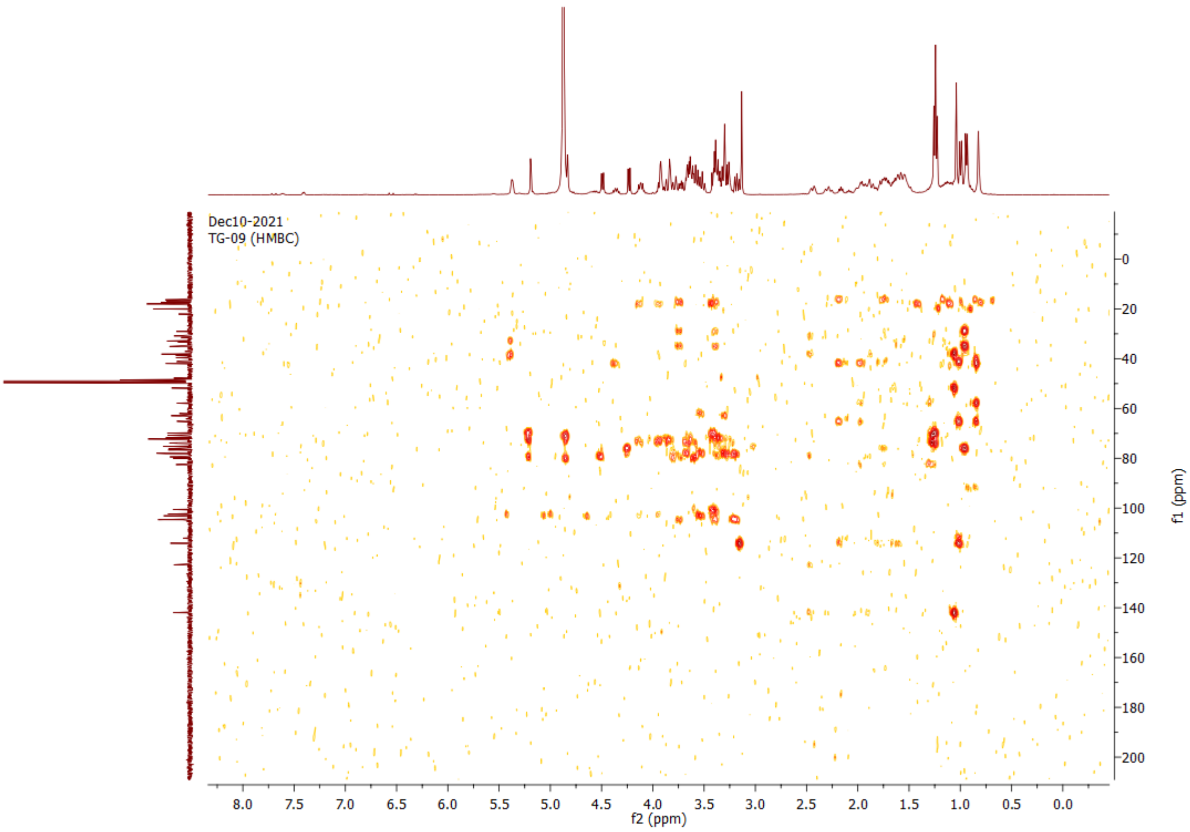

Figure S14. HMBC Spectra of compound TG 09

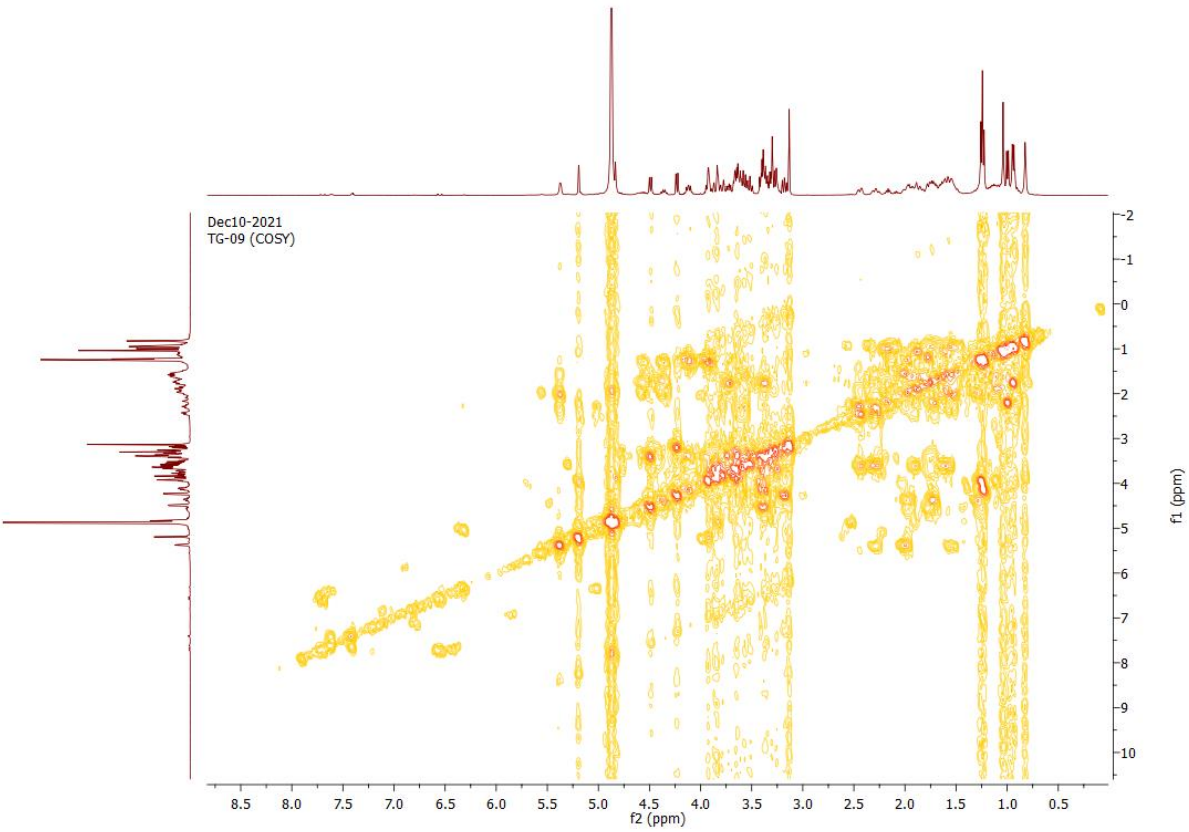

Figure S15. COSY Spectra of compound TG 09

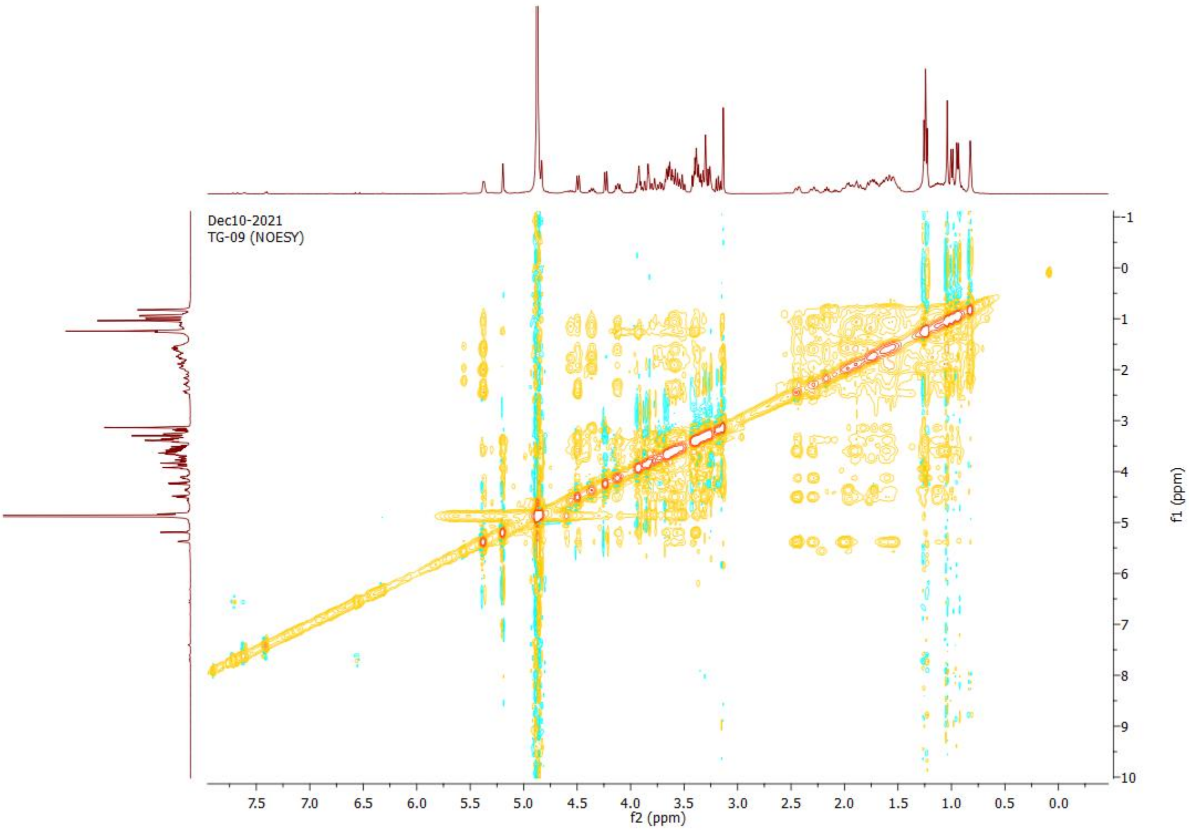

Figure S16. NOESY Spectra of compound TG 09

ental Composition Report

Page 1

gle Mass Analysis

olerance = 50.0 PPM / DBE: min = -1.5, max = 50.0
Element prediction: Off
Number of isotope peaks used for i-FIT = 3

Monoisotopic Mass, Even Electron Ions
23 formula(e) evaluated with 1 results within limits (up to 3 closest results for each mass)
Elements Used:
C: 0-51 H: 0-100 O: 0-22

TG-09 QMI DIVISION, CSIR-IIIM JAMMU
Xevo G2-XS QTOF YFC2015

05-Dec-2022
13:06:41
1: TOF MS ES+
1.07e+006

051222_21 9 (0.209)

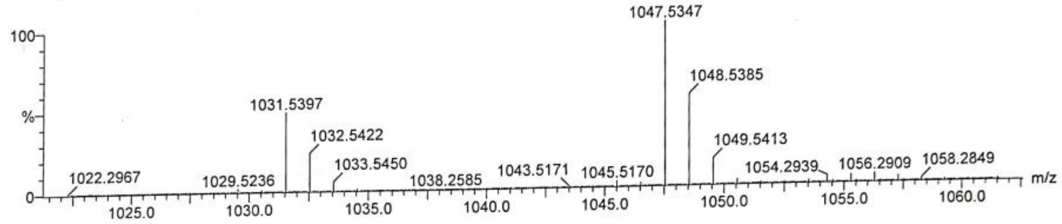

Minimum:				-1.5				
Maximum:		2.0	50.0	50.0				
Mass	Calc. Mass	mDa	PPM	DBE	i-FIT	Norm	Conf(%)	Formula
1049.5413	1049.5532	-11.9	-11.3	9.5	550.5	n/a	n/a	C51 H85 O22

Figure S17. HR-ESIMS of compound TG 09

IIIM GCMS ANALYSIS REPORT

Sample Information

**Analyzed by**	**: ADMIN**
**Analyzed**	**: 1/26/2022 11:14:29 PM**
**Sample Type**	**: Unknown**
**Sample Name**	**: TG-07(B3)**
**Sample Amount**	**: 1**
**Vial #**	**: 3**
**Injection Volume**	**: 0.50**
**Data File**	**: D:\GCMS DATA\LIQUID\JAN 2022\25.01.2022\25.01.2022 BHARTI034.qgd**
**Method File**	**: D:\GCMS METHOD\GCMS-GENERAL AMIT 1.qgm**
**Tuning File**	**: D:\Tuning\Tuning-NM-F1 18.01.2022.qgt**
**Modified by**	**: Admin**
**Modified**	**: 1/26/2022 11:59:21 PM**

Chromatogram TG-07(B3) D:\GCMS DATA\LIQUID\JAN 2022\25.01.2022\25.01.2022 BHARTI034.qgd

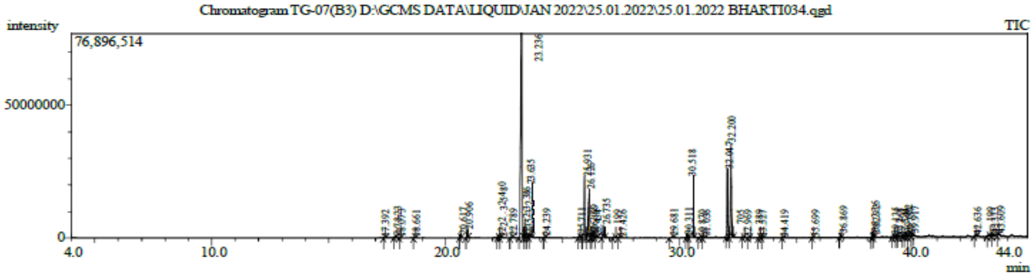

Peak Report TIC

Peak#	R.Time	Area	Area%	Height	Similarity	Name
1	23.215	231224721	28.46	76755223	93	Acetyl 2,3,4-tri-O-acetyl-6-deoxy-.alpha.-D-glucopyranosid
2	23.635	55669651	6.85	18773884	95	alpha.-l-Talopyranoside, methyl 6-deoxy-, triacetate
3	25.931	84146429	10.36	23296999	95	Methyl 2,3,4-tri-O-acetyl-6-deoxy-.alpha.-D-glucopyranosid
4	26.126	63098359	7.77	18205988	93	Acetyl 2,3,4-tri-O-acetyl-6-deoxy-.beta.-D-glucopyranoside
5	26.202	4369319	13.54	1550231	88	Tetraacetyl .beta.-l-rhamnose
6	25.530	3965918	0.49	1363282	94	alpha.-D-Xylopyranose, tetraacetate
7	32.047	71127361	18.75	25857099	95	beta.-D-Galactopyranose, pentaacetate
8	32.200	99974618	12.30	35119152	93	beta.-D-Glucopyranose pentaacetate

Figure S18. GC/MS analysis of compound TG 07 B3

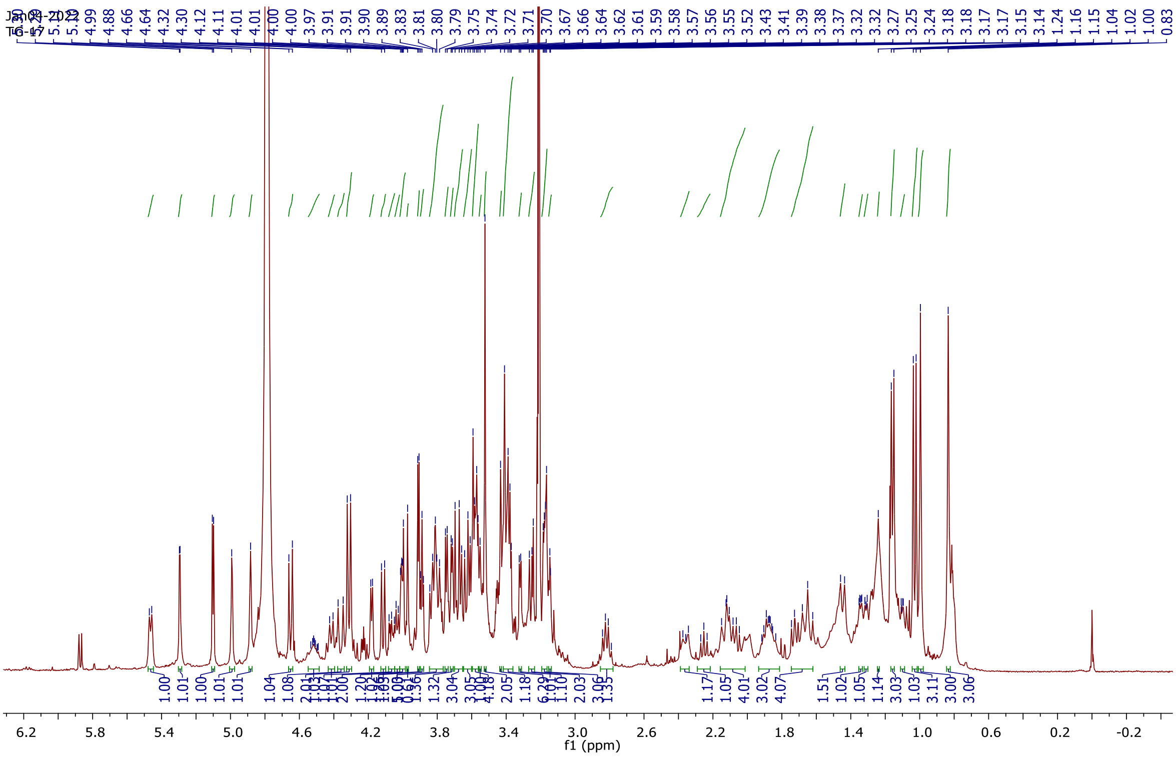

Figure S19. ^{1}HNMR spectrum of compound **3 (TG-12)** (CD_3OD, 500 MHz)

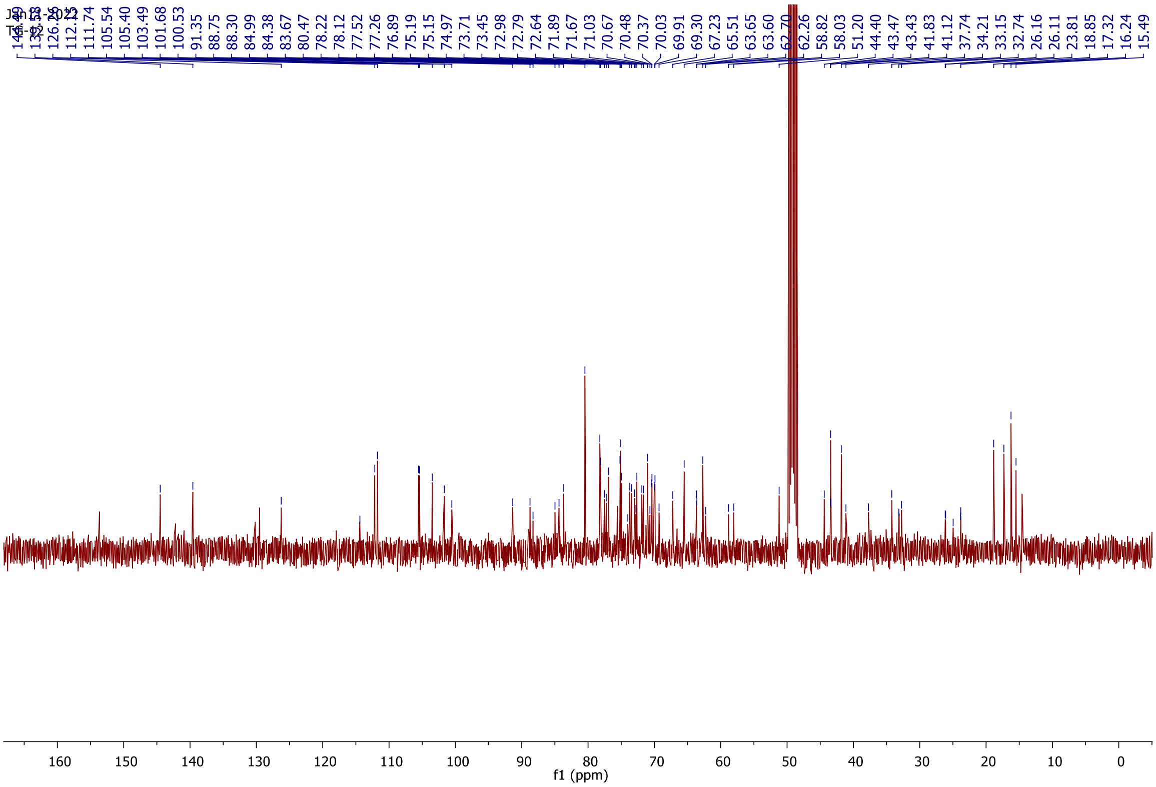

Figure S20.^{13}C NMR spectrum of compound **3** (CD_3OD, 100 MHz)

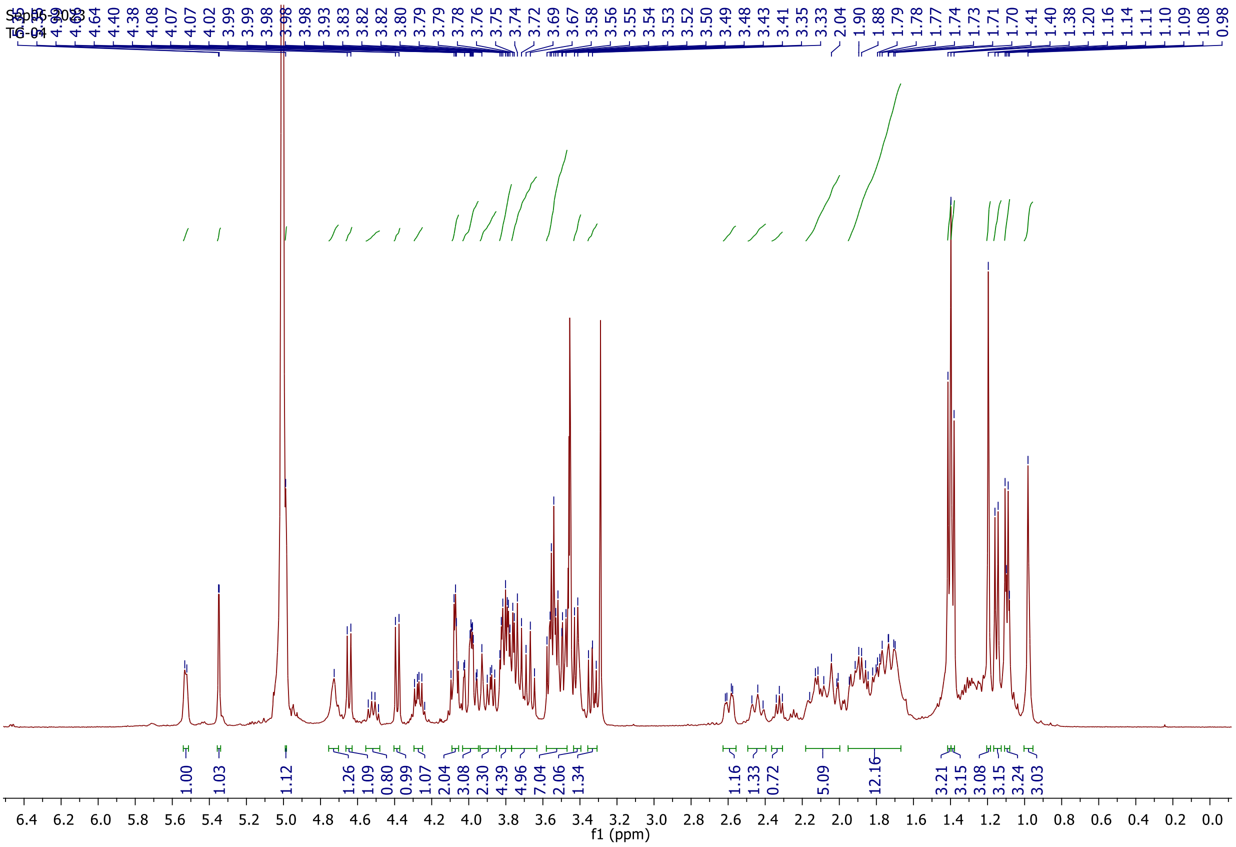

Figure S21. ^{1}HNMR spectrum of compound **4 (TG-04)** (CD_3OD, 500 MHz)

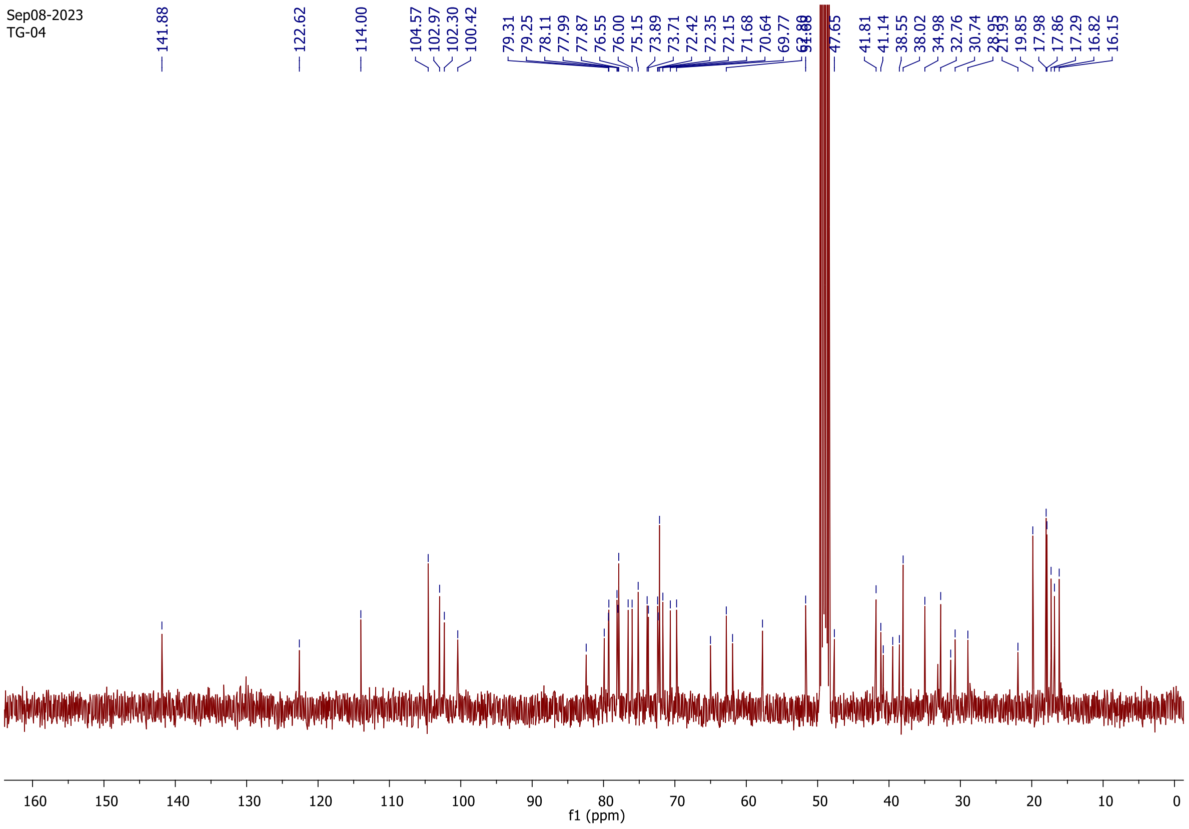

Figure S22.^{13}C NMR spectrum of compound **4** (CD_3OD, 100 MHz)

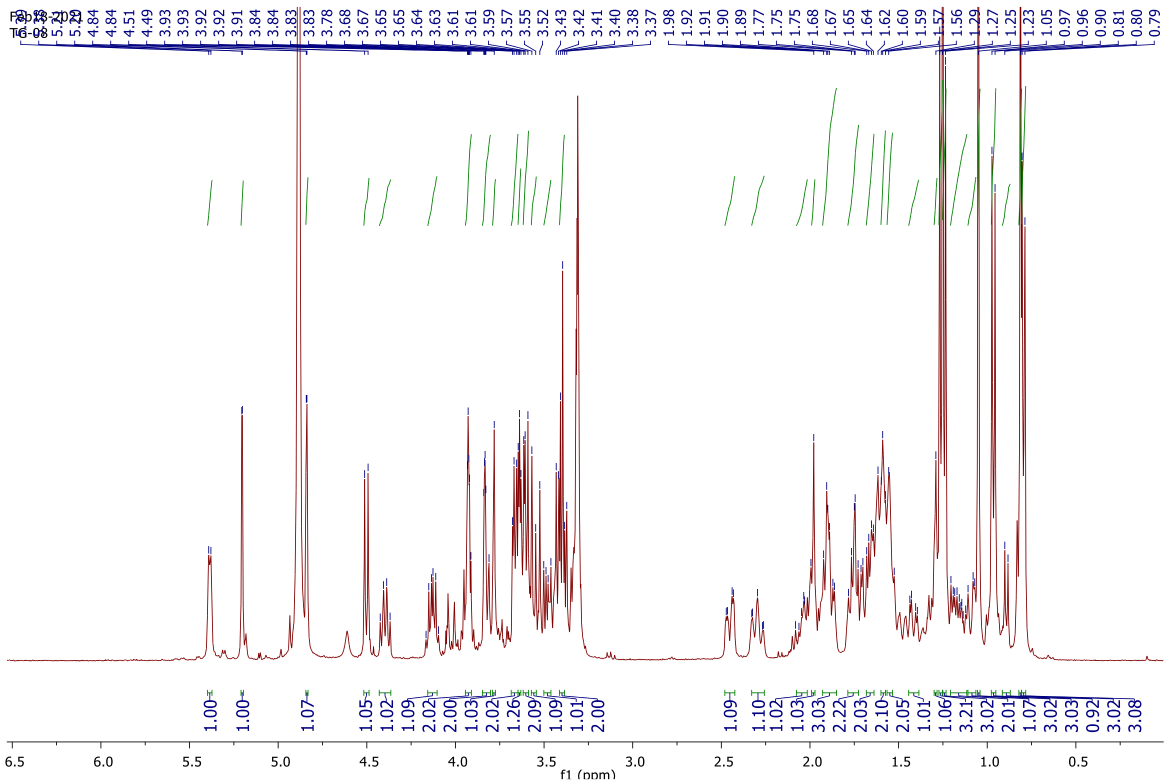

Figure S23. ^{1}HNMR spectrum of compound **5 (TG-08)** (CD_3OD, 500 MHz)

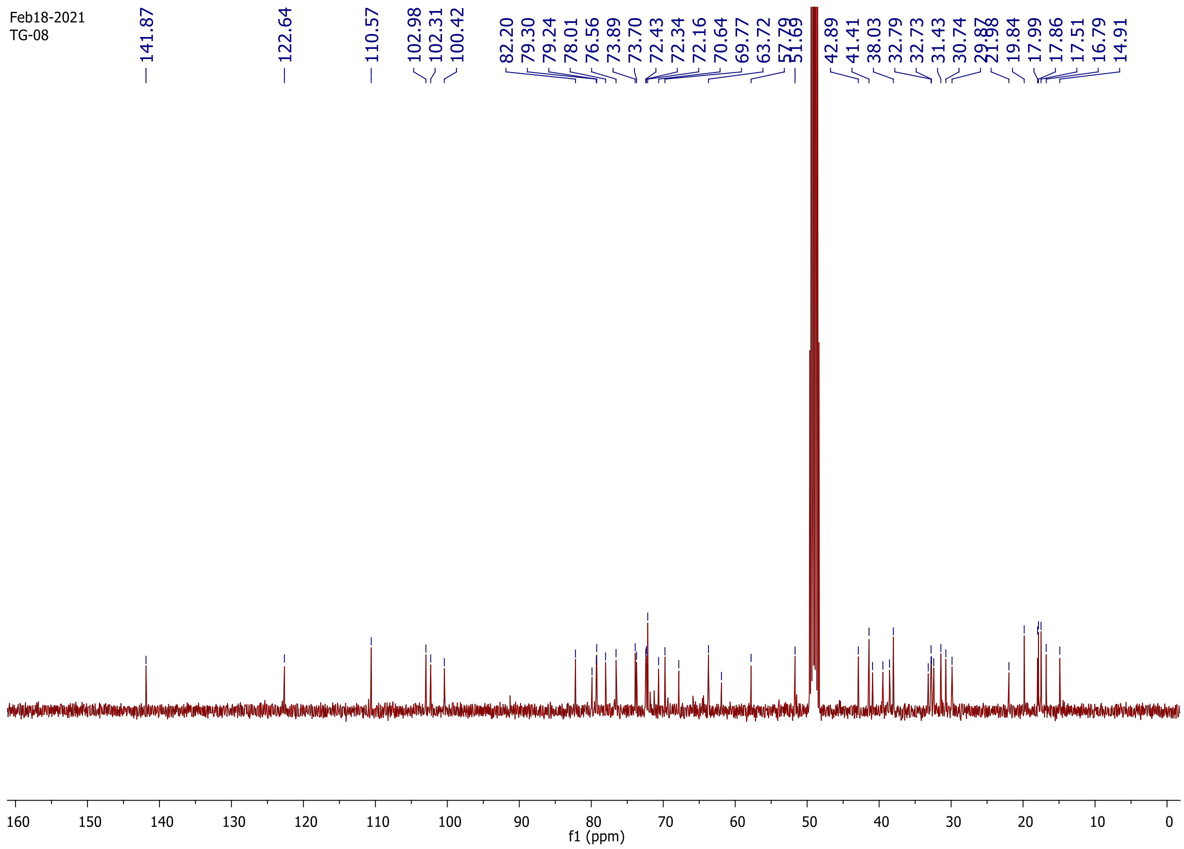

Figure S24.^{13}C NMR spectrum of compound **5** (CD_3OD, 100 MHz)

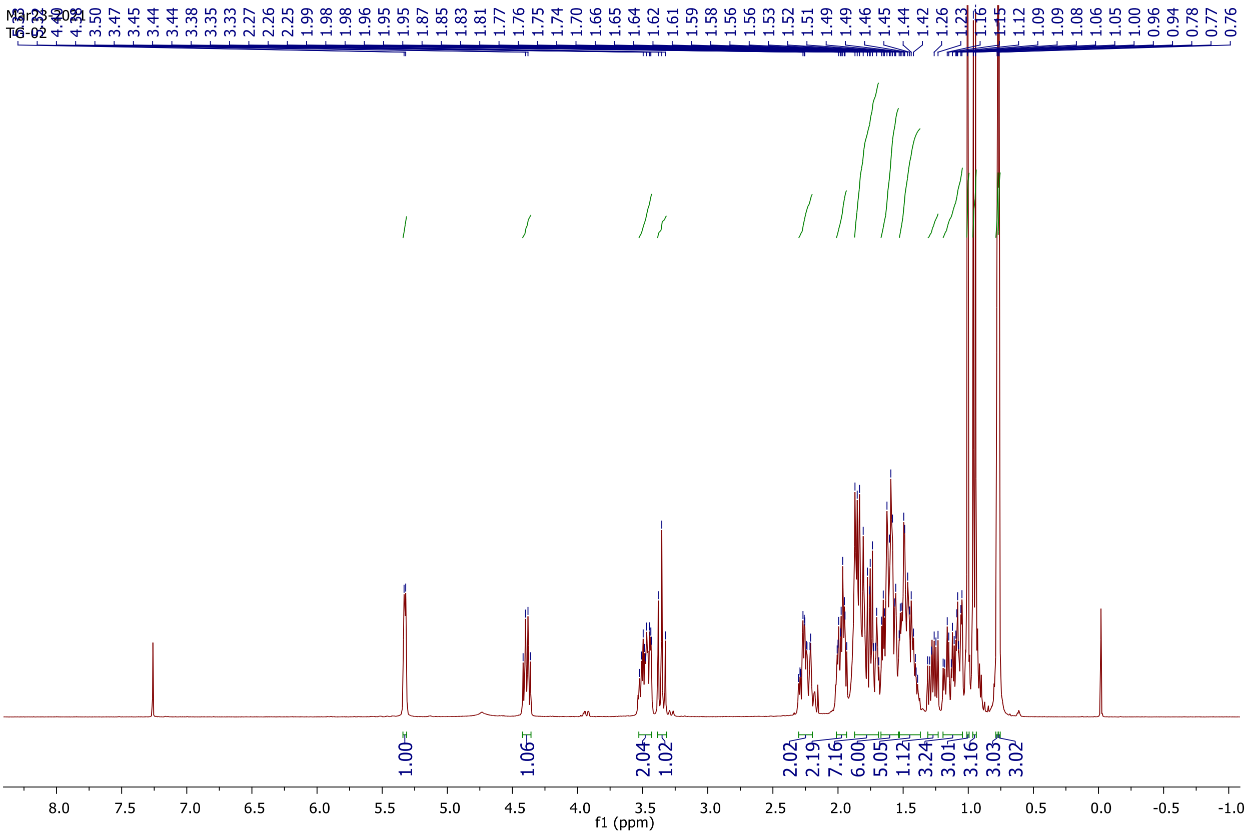

Figure S25. ^{1}HNMR spectrum of compound **6 (TG-02)** (CD_3OD, 500 MHz)

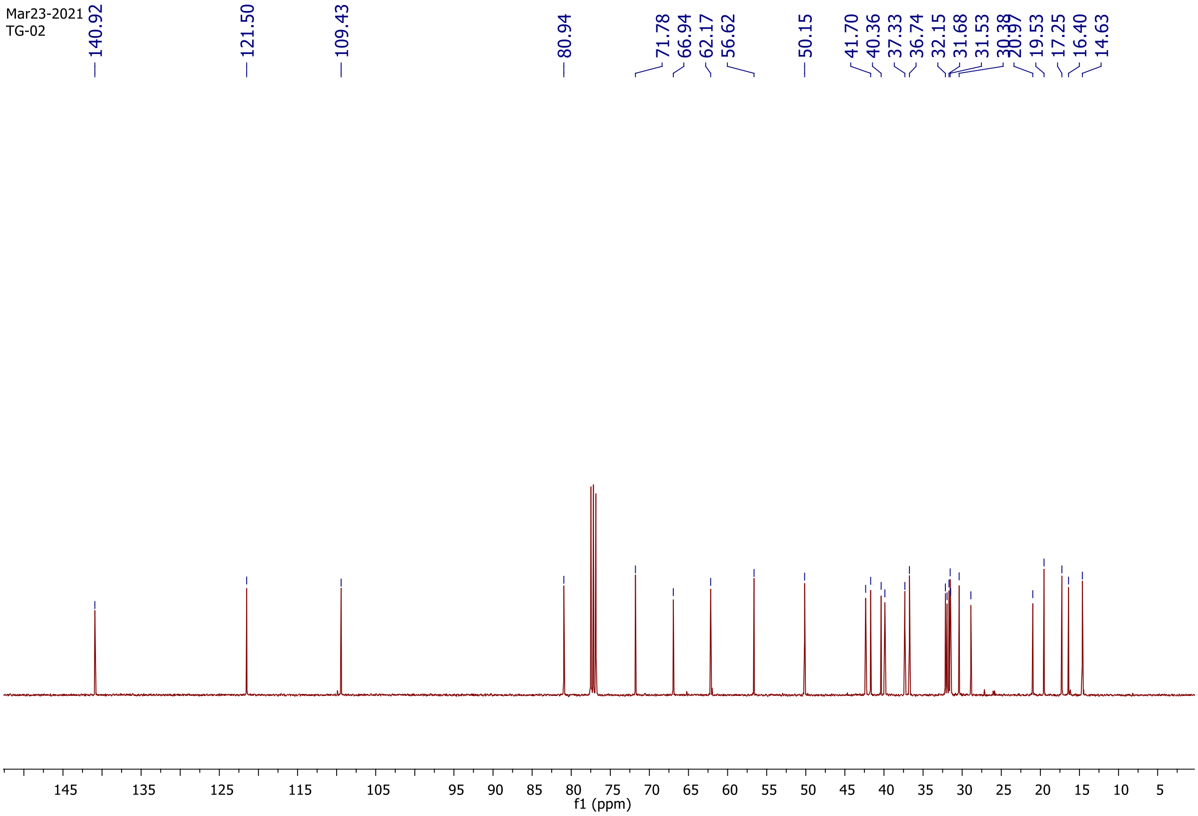

Figure S26.^{13}C NMR spectrum of compound **6** (CD_3OD, 100 MHz)

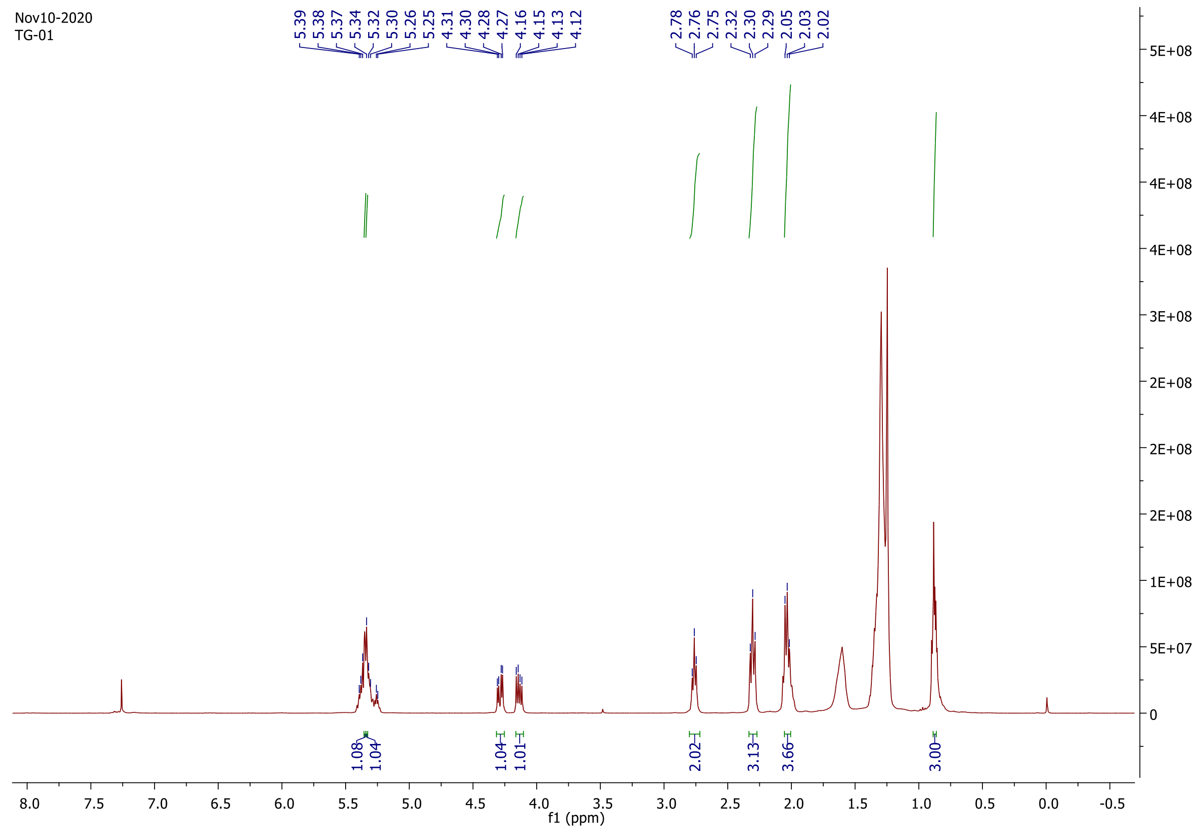

Figure S27. ^{1}HNMR spectrum of compound **7 (TG-02)** ($CDCl_3$, 500 MHz)

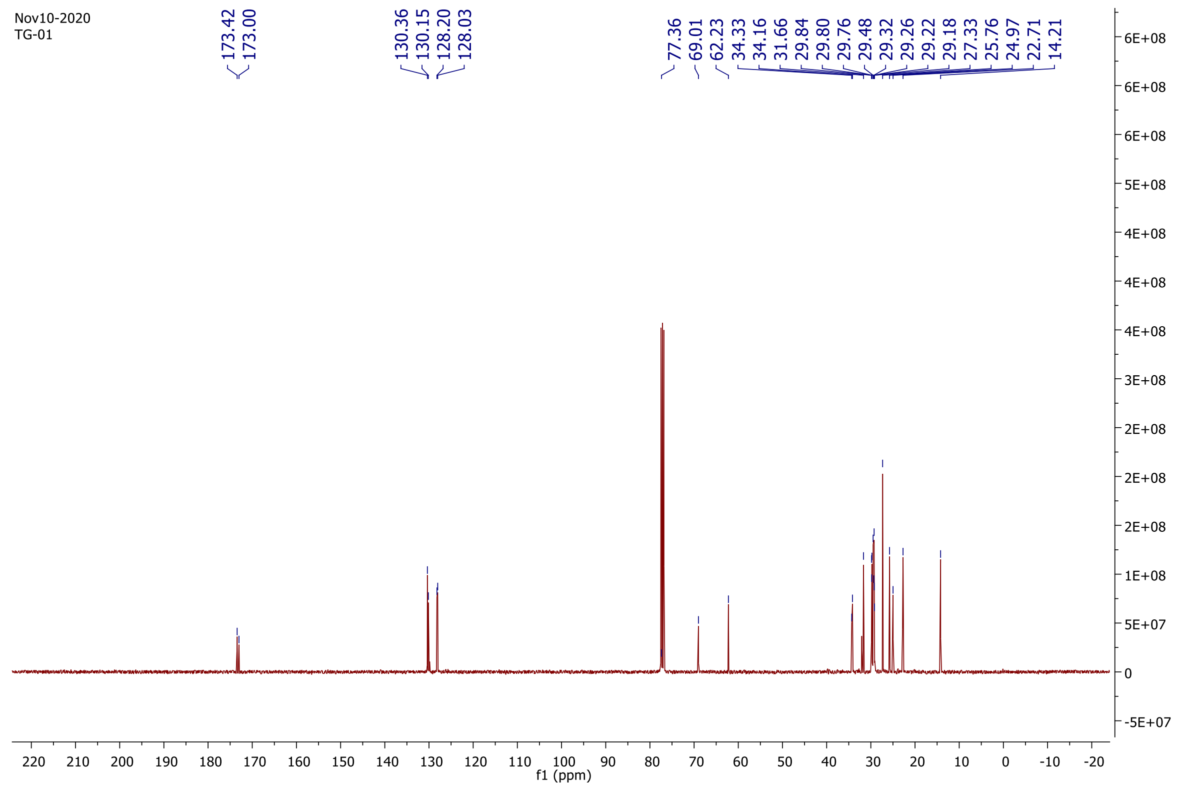

Figure S28.^{13}C NMR spectrum of compound **7** ($CDCl_3$, 100 MHz)

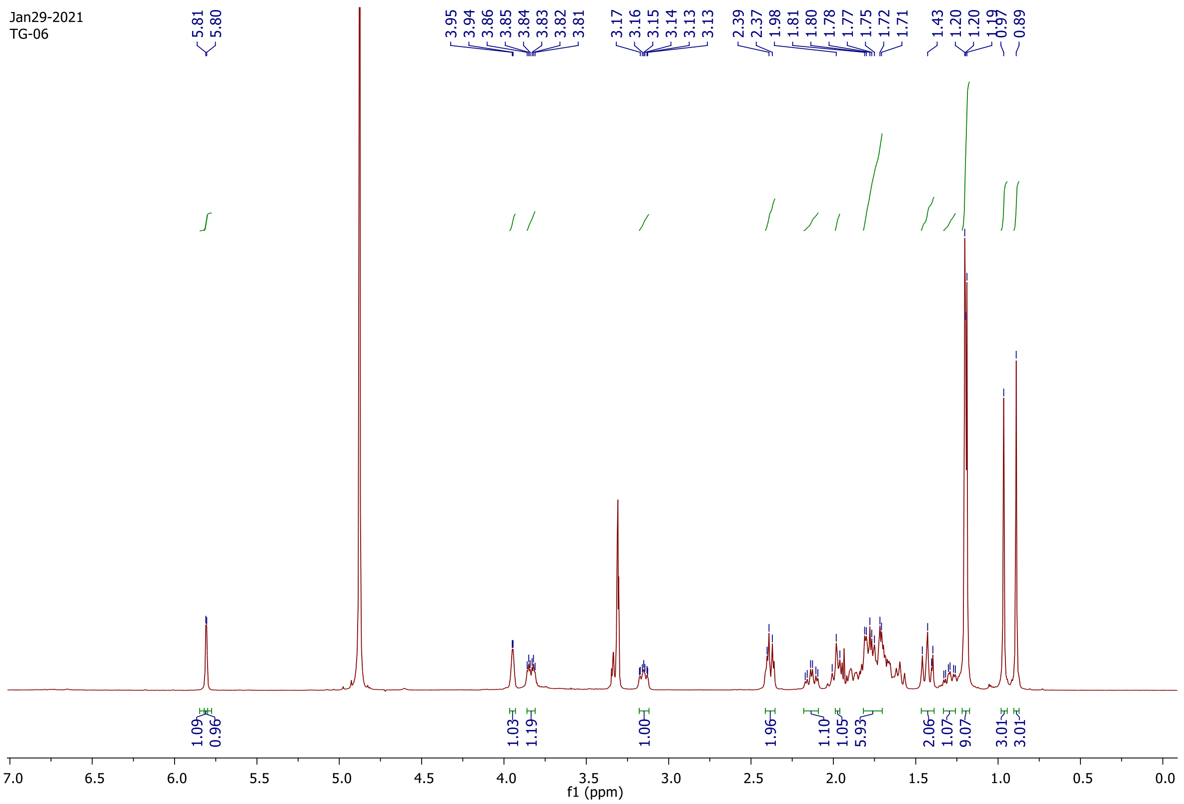

Figure S29. ^{1}HNMR spectrum of compound **8 (TG-05)** (CD_3OD, 500 MHz)

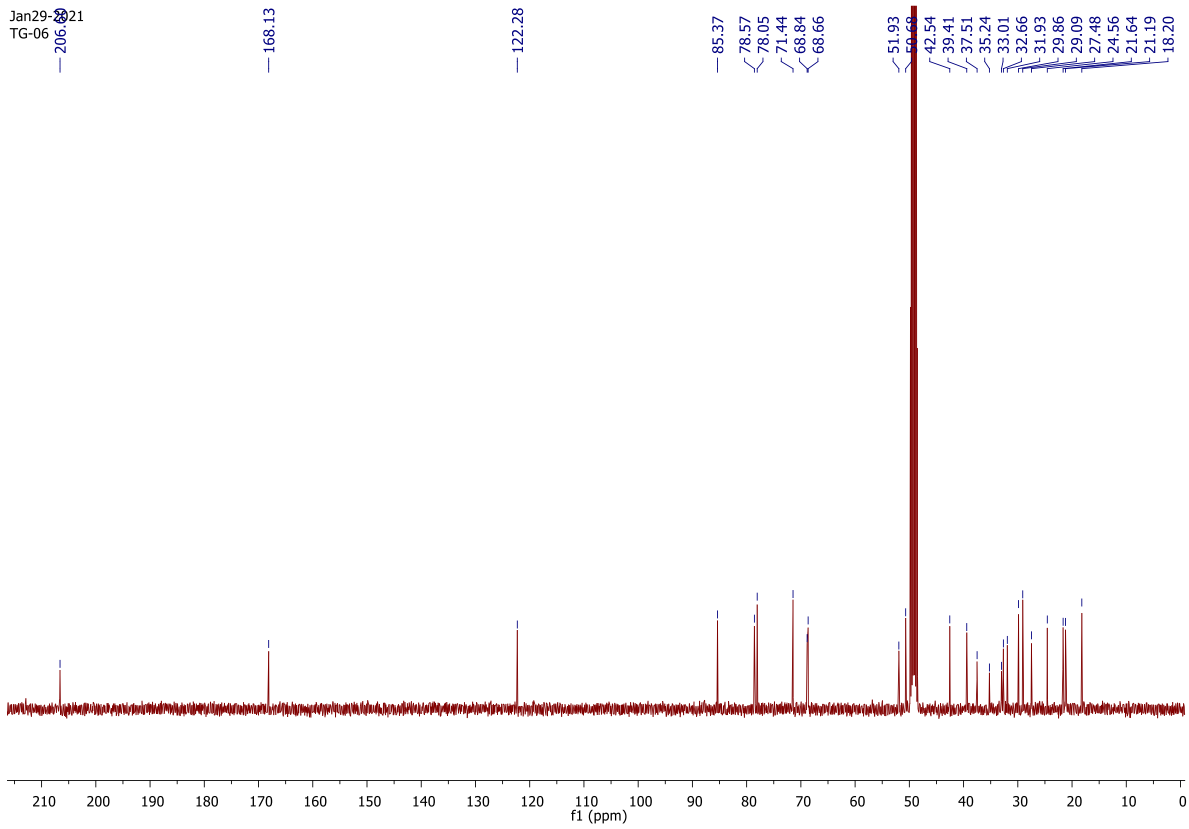

Figure S30.^{13}C NMR spectrum of compound **8** ($MeOD_6$, 100 MHz)

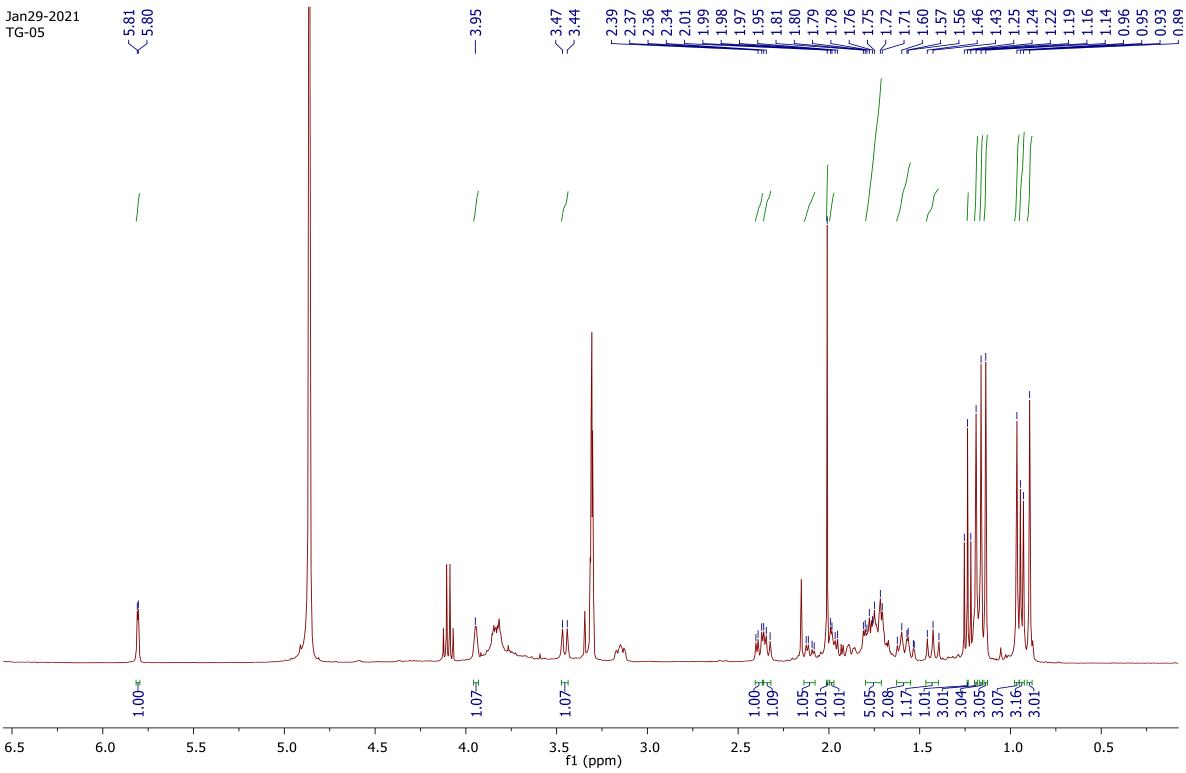

Figure S31. ^{1}HNMR spectrum of compound **9 (TG-06)** (CD_3OD, 500 MHz)

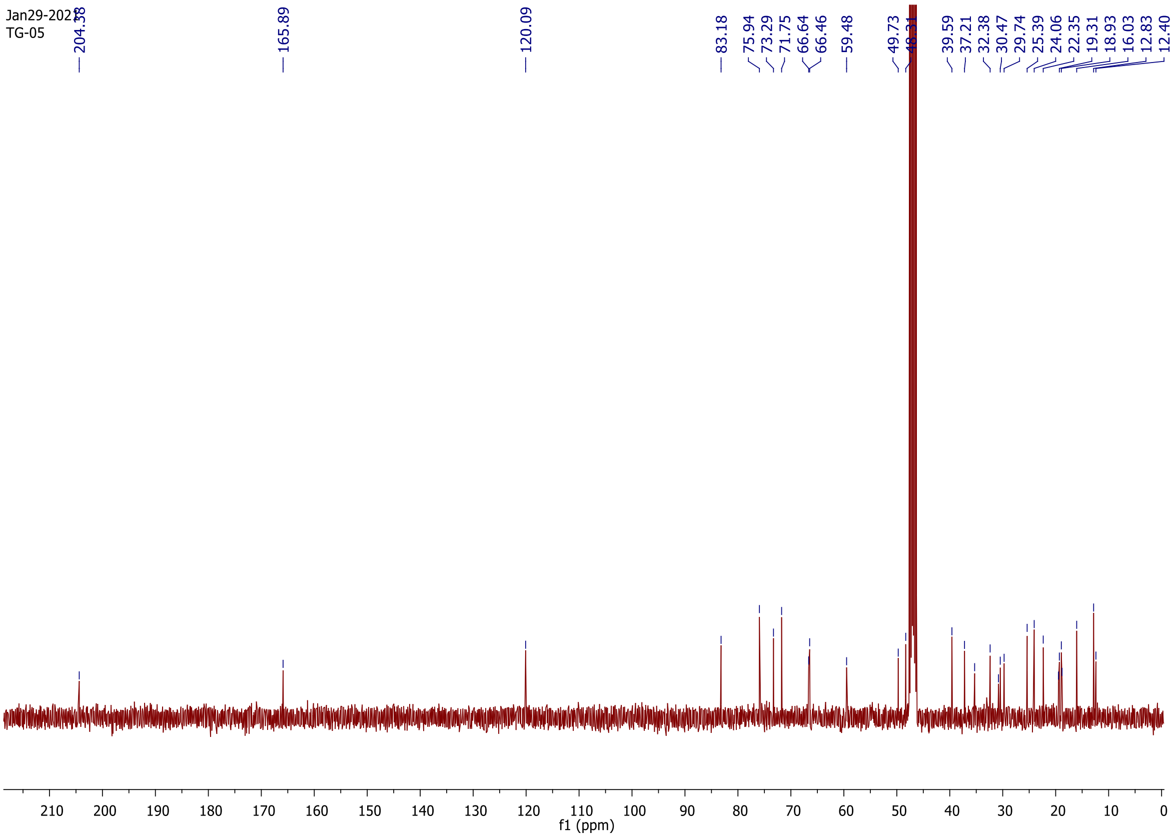

Figure S32.^{13}C NMR spectrum of compound **9** (CD_3OD, 100 MHz)

Table 1: Growth inhibitory effect of Extracts and enriched fractions against different cancer cell lines

Extract & Fractions	Conc. (µg/mL)	Human cancer cell lines						
		**Breast**	**Breast**	**Colon**	**Colon**	**Lung**	**Pancreatic**	**Prostate**
		**MCF-7**	**MDA MB-231**	**HCT-116**	**SW-620**	**A-549**	**MIA PaCa-2**	**PC-3**
		**Growth Inhibition (%)**						
**TG-E1**	**100**	16.88	28.55	11.83	10.97	12.49	**ND**	**ND**
	**50**	5.24	11.87	8.39	3.22	4.56	**ND**	**ND**
**TG-E3**	**100**	48.22	40.28	72.91	**72.50**	**66.46**	30.87	67.75
	**50**	23.07	18.76	50.73	58.87	35.07	15.74	52.26
**TG-EF**	**100**	60.22	58.22	86.66	**91.29**	**96.30**	68.87	85.99
	**50**	45.40	34.78	76.77	75.39	82.78	42.48	66.55

Table 2: SRB assay-based screening results. **IC_{50} values (μM) of compounds on panel of different human cancer cell lines and normal cell line.

**IC_{50} (μM)± SD**										
Compd	A-549	HCT-116	MCF-7	MDA-MB 231	Mia PaCa-2	SW-620	PC-3	SHSY-5Y	HOP-62	fR2
1	1.83± 0.54	2.91± 0.50	4.40± 0.33	1.90± 0.26	1.94± 0.24	1.85± 0.52	3.18 ± 0.37	2.43 ± 0.32	1.92± 0.62	13.75 ±0.42
2	1.79± 0.47	3.47± 0.39	5.92± 0.55	9.49± 0.25	-	3.18± 0.40	4.97 ± 0.58	-	-	10.84 ±0.35
3	>50	>50	>50	>50	>50	>50	>50	>50	>50	>50
4	2.08± 0.56	1.95± 0.45	6.98± 0.39	8.29± 0.33	3.79± 0.49	1.98± 0.46	3.77 ± 0.52	3.33 ± 0.26	2.65± 0.47	-
5	1.50± 0.62	4.44± 0.57	4.15± 0.47	2.56± 0.33	1.69 ± 0.45	1.66± 0.38	1.85 ± 0.43	4.43 ± 0.44	1.85± 0.57	2.71± 0.23
6	>50	>50	>50	>50	>50	>50	>50	>50	>50	-
7	30.46 ± 0.49	>50	29.40 ± 0.37	>50	>50	>50	>50	>50	>50	-
8	>50	>50	>50	>50	>50	>50	>50	>50	>50	-
9	32.95 ± 0.43	>50	38.99 ± 0.63	>50	33.26± 0.57	>50	>50	>50	>50	-
**CPT**	0.030 ±0.04	0.160± 0.23	0.147 ±0.13	0.125 ±0.03	0.060± 0.02	0.093 ±0.03	0.170±0.03	-	0.065 ±0.04	-

References

1. Sofi, I.I., Verma, S., Charles, B., Ganie, A. H., Sharma, N., & Shah, M. A. 2022. Predicting distribution and range dynamics of *Trillium govanianum* under climate change and growing human footprint for targeted conservation. Plant Ecology. 223, 1-17. https://doi.org/10.1007/s11258-021-01189-3

2. Ohara, M., Kawano, S., 2005. Life-history monographs of Japanese plants. 2: *Trillium camschatcense* Ker-Gawl (Trilliaceae). Plant Species Biol. 20, 75–82. https://doi.org/10.1111/j.1442-1984.2005.00126.x.

3. Chauhan, H.K., Bisht, A.K., Bhatt, I.D., Bhatt, A., Gallacher, D., 2019. *Trillium*-toward sustainable utilization of a biologically distinct genus valued for traditional medicine, Bot. Rev. 85, 252–272. https://doi.org/10.1007/s12229-019-09211-0

4. Sharma, P., Samant, S., 2104. Diversity distribution and indigenous uses of medicinal plants in Parbati valley of Kullu district in Himachal Pradesh, North-western Himalaya, Asian. J. Adv. Basic Sci. 2, 77–98. https://www.jmbm.in/index.php/jmbas/article/view/6

5. Pant, S., Samant, S.S., 2010. Ethnobotanical observations in the Mornaula reserve forest of Komoun, West Himalaya, India. Ethnobot. Leafl. 2010,8. https://opensiuc.lib.siu.edu/ebl/vol2010/iss2/8

6. Rani, S., Rana, J.C., & Rana, P.K., 2013. Ethnomedicinal plants of Chamba district, Himachal Pradesh, India. J. Med. Plant Res. 7, 3147-3157. DOI:10.5897/JMPR2013.5249

7. Sharma, O.R., Arya, D., Goel, S., Vyas, K., Shinde, P., 2018. *Trillium govanianum* Wall. ex D. Don (Nagchatri): an important ethnomedicinal plant of Himalayan region (Himachal Pradesh). J. Med. Plants Stud. 6, 11-13. ISSN (E): 2320-3862

8. Ono, M., Hamada, T., Nohara, T., 1986. An 18-norspirostanol glycoside from *Trillium tschonoskii*. Phytochemistry, 25, 544-545. https://doi.org/10.1016/S0031-9422(00)85524-7

9. Mimaki, Y., Watanabe, K., 2008. Clintoniosides A–C, new polyhydroxylated spirostanol glycosides from the rhizomes of *Clintonia udensis*. Helv. Chim. Acta. 91, 2097-2106. https://doi.org/10.1002/hlca.200890224

10. Huang, W., & Zou, K., 2011. Cytotoxicity of a plant steroidal saponin on human lung cancer cells. Asian Pac. J. Cancer Prev, 12(2), 513-517.

11. Ur Rahman, S., Ismail, M., Khurram, M., Ullah, I., Rabbi, F., Iriti, M., 2017. Bioactive steroids and saponins of the genus Trillium. Molecules. 22, 2156. https://doi.org/10.3390/molecules22122156

12. Yan, T., Wang, A., Hu, G., Jia, J., 2021. Chemical constituents of *Trillium tschonoskii* Maxim. Nat. Prod. Res. 35, 3351-3359. https://doi.org/10.1080/14786419.2019.1700245

13. Yan, L.L., Zhang, Y.J., Gao, W.Y., Man, S.L., Wang, Y., 2009. In vitro and in vivo anticancer activity of steroid saponins of *Paris polyphylla* var. yunnanensis. Exp. Oncol. 27-32. http://dspace.nbuv.gov.ua/handle/123456789/135101

14. Ya-Zheng, Z.H.A.O., Yuan-Yuan Zhang, H.A.N., Han, F.A.N., Rui-Ping, H.U., Yang, Liang Zhong, K.O.U., Jun-Ping, K.O.U., Bo-Yang, Y.U., 2018. Advances in the antitumor activities and mechanisms of action of steroidal saponins. Chin. J. Nat. Med. 16, 732-748. https://doi.org/10.1016/S1875-5364(18)30113-4.
15. Ur Rahman, S., Adhikari, A., Ismail, M., Raza Shah, M., Khurram, M., Shahid, M., Ali, F., Haseeb, A., Akbar, F., Iriti, M., 2016. Beneficial effects of *Trillium govanianum* rhizomes in pain and inflammation. Molecules. 21, 1095. https://doi.org/10.3390/molecules21081095
16. Ismail, M., Shah, M.R., Adhikari, A., Anis, I., Ahmad, M.S., Khurram, M., 2015. Govanoside A, a new steroidal saponin from rhizomes of *Trillium govanianum*. Steroids, 104, 270-275. https://doi.org/10.1016/j.steroids.2015.10.013
17. Singh, P.P., Suresh, P.S., Bora, P.S., Bhatt, V., Sharma, U., 2021. Govanoside B, a new steroidal saponin from rhizomes of *Trillium govanianum*. Nat. Prod. Res. 36, 37-45. https://doi.org/10.1080/14786419.2020.1761360
18. Abdel-Sattar, E., Shabana, M.M., El-Mekkawy, S., 2008. Protodioscin and pseudoprotodioscin from *Solanum intrusum*. Res. J. Phytochem. 2, 100-105.
19. Yokosuka, A., Mimaki, Y., 2008. Steroidal glycosides from the underground parts of *Trillium erectum* and their cytotoxic activity. Phytochem. 69, 2724-2730. https://doi.org/10.1016/j.phytochem.2008.08.004
20. Love, J., Simons, C.R., 2020. Acid hydrolysis of saponins extracted in tincture. PloS one 15, e0244654. https://doi.org/10.1371/journal.pone.0244654
21. Desai, A.G., Qazi, G.N., Ganju, R.K., El-Tamer, M., Singh, J., Saxena, A.K., Bedi, Y.S., Taneja, S.C., Bhat, H.K., 2008. Medicinal plants and cancer chemoprevention. Curr. Drug Metab. 9, 581-591. https://doi.org/10.2174/138920008785821657
22. Vichai, V., Kirtikara, K., 2006. Sulforhodamine B colorimetric assay for cytotoxicity screening. Nat. Protoc. 1, 1112-1116. https://doi.org/10.1038/nprot.2006.179
23. Gupta, N., Rath, S.K., Singh, J., Qayum, A., Singh, S., Sangwan, P.L., 2017. Synthesis of novel benzylidene analogues of betulinic acid as potent cytotoxic agents. Eur. J. Med. Chem. 135, 517-530. https://doi.org/10.1016/j.ejmech.2017.04.062

24. Gupta, N., Sharma, S., Raina, A., Bhushan, S., Malik, F.A., Sangwan, P.L., 2017. Synthesis of Novel Mannich Derivatives of Bakuchiol as Apoptotic Inducer through Caspase Activation and PARP-1 Cleavage in A549 Cells. ChemistrySelect. 2, 5196-5201. https://doi.org/10.1002/slct.201700504

25. Perillo, B., Di Donato, M., Pezone, A., Di Zazzo, E., Giovannelli, P., Galasso, G., Castoria, G. Migliaccio, A., 2020. ROS in cancer therapy: The bright side of the moon. Exp. Mol. Med. 52, 192-203. https://doi.org/10.1038/s12276-020-0384-2
26. Ju, Y., Jia, Z.J., 1992. Steroidal saponins from the rhizomes of *Smilax menispermoidea*. Phytochem. 31, 1349-1351. https://doi.org/10.1016/0031-9422(92)80288-P
27. Mimaki, Y., Aoki, T., Jitsuno, M., Kiliç, C.S., Coşkun, M., 2008. Steroidal glycosides from the rhizomes of *Ruscus hypophyllum*. Phytochem. 69, 729-737. https://doi.org/10.1016/j.phytochem.2007.09.022
28. Braca, A., Prieto, J.M., De Tommasi, N., Tomè, F., Morelli, I., 2004. Furostanol saponins and quercetin glycosides from the leaves of *Helleborus viridis* L. Phytochem. 65, 2921-2928. https://doi.org/10.1016/j.phytochem.2004.07.013
29. Elmore, S., 2007 Apoptosis: a review of programmed cell death. Toxicol. Pathol. 35, 495 -516. https://doi.org/10.1080/01926230701320337
30. Biancardi, A., Biver, T., Secco, F., Mennucci, B., 2013. An investigation of the photophysical properties of minor groove bound and intercalated DAPI through quantum-mechanical and spectroscopic tools. Phys. Chem. Chem. Phys. 15, 4596-4603. https://doi.org/10.1039/C3CP44058C
31. Pathania, A.S., Wani, Z.A., Guru, S.K., Kumar, S., Bhushan, S., Korkaya, H., Seals, D.F., Kumar, A., Mondhe, D.M., Ahmed, Z., Chandan, B.K., Malik, F., 2015. The anti-angiogenic and cytotoxic effects of the boswellic acid analog BA145 are potentiated by autophagy inhibitors. Mol. Cancer. 14, 1-15. https://doi.org/10.1186/1476-4598-14-6
32. Perillo, B., Di Donato, M., Pezone, A., DiZazzo, E., Giovannelli, P., Galasso, G., 2020. ROS in cancer therapy: The bright side of the moon, Exp. Mol. Med. 52, 192-203. https://doi.org/10.1038/s12276-020-0384-2
33. Gottlieb, E., Armour, S.M., Harris, M.H., Thompson, C.B., 2003. Mitochondrial membrane potential regulates matrix configuration and cytochrome c release during apoptosis. Cell Death Differ. 10, 709-717. https://doi.org/10.1038/sj.cdd.4401231
34. Kumar, A., Singh, B., Sharma, P.R., Bharate, S.B., Saxena, A.K., Mondhe, D.M., 2016. A novel microtubule depolymerizing colchicine analogue triggers apoptosis and autophagy in HCT-116 colon cancer cells. Cell Biochem. Funct. 34, 69-81. https://doi.org/10.1002/cbf.3166

35. Rodriguez, L.G., Wu, X., Guan, J.L., 2005. Wound-healing assay. Cell Migration: Developmental Methods and Protocols, vol 294. Humana Press. 23-29. https://doi.org/10.1385/1-59259-860-9:023
36. Munshi, A., Hobbs, M., Meyn, R.E., 2005. Clonogenic cell survival assay. In: Blumenthal, R.D. (eds) Chemosensitivity: Methods in Molecular Medicine, vol 110. Humana Press. 21-28. https://doi.org/10.1385/1-59259-869-2:021
37. Majeed, R., Hamid, A., Sangwan, P.L., Chinthakindi, P.K., Koul, S., Rayees, S., Singh, G., Mondhe, D.M., Mintoo, M.J., Singh, S.K., Rath, S.K., Saxena, A.K., 2014. Inhibition of phosphotidylinositol-3 kinase pathway by a novel naphthol derivative of betulinic acid induces cell cycle arrest and apoptosis in cancer cells of different origin. Cell Death Dis. 5, e1459-e1459. https://doi.org/10.1038/cddis.2014.387
